# Conditional Cash Transfer Program and Leprosy Incidence: Analysis of 12.9 Million Families From the 100 Million Brazilian Cohort

**DOI:** 10.1093/aje/kwaa127

**Published:** 2020-07-08

**Authors:** Julia M Pescarini, Elizabeth Williamson, Maria Y Ichihara, Rosemeire L Fiaccone, Laura Forastiere, Anna Ramond, Joilda Silva Nery, Maria Lucia F Penna, Agostino Strina, Sandra Reis, Liam Smeeth, Laura C Rodrigues, Elizabeth B Brickley, Gerson O Penna, Mauricio L Barreto

**Keywords:** Bolsa Familia Program, cash transfers, Hansen’s disease, inequality, infectious diseases, poverty

## Abstract

Leprosy is a neglected tropical disease predominately affecting poor and marginalized populations. To test the hypothesis that poverty-alleviating policies might be associated with reduced leprosy incidence, we evaluated the association between the Brazilian Bolsa Familia (BFP) conditional cash transfer program and new leprosy case detection using linked records from 12,949,730 families in the 100 Million Brazilian Cohort (2007–2014). After propensity score matching BFP beneficiary to nonbeneficiary families, we used Mantel-Haenszel tests and Poisson regressions to estimate incidence rate ratios for new leprosy case detection and secondary endpoints related to operational classification and leprosy-associated disabilities at diagnosis. Overall, cumulative leprosy incidence was 17.4/100,000 person-years at risk (95% CI: 17.1, 17.7) and markedly higher in “priority” (high-burden) versus “nonpriority” (low-burden) municipalities (22.8/100,000 person-years at risk, 95% confidence interval (CI): 22.2, 23.3, compared with 14.3/100,000 person-years at risk, 95% CI: 14.0, 14.7). After matching, BFP participation was not associated with leprosy incidence overall (incidence rate ratio (IRR)_Poisson_ = 0.97, 95% CI: 0.90, 1.04) but was associated with lower leprosy incidence when restricted to families living in high-burden municipalities (IRR_Poisson_ = 0.86, 95% CI: 0.77, 0.96). In high-burden municipalities, the association was particularly pronounced for paucibacillary cases (IRR_Poisson_ = 0.82, 95% CI: 0.68, 0.98) and cases with leprosy-associated disabilities (IRR_Poisson_ = 0.79, 95% CI: 0.65, 0.97). These findings provide policy-relevant evidence that social policies might contribute to ongoing leprosy control efforts in high-burden communities.

## Abbreviations

BRL: Brazilian reals
BFP: Bolsa Familia Program
CadUnico: Brazilian National Registry for Social Programs Cadastro Único
CCT: conditional cash-transfers
CI: confidence interval
IRR: incidence rate ratio
MB: multibacillary
MH: Mantel-Haenszel
PB: paucibacillary
PS: propensity score
SINAN: Brazilian Notifiable Disease Registry

Leprosy is a neglected tropical disease that can lead to blindness and permanent disabilities if left untreated. While the prevalence of leprosy has declined over the last 30 years, leprosy continues to be an important cause of disability and stigma among the over 200,000 individuals diagnosed annually worldwide (1, 2). There is an increasing recognition that leprosy and other neglected tropical diseases are strongly linked to poverty, being both attributable to and responsible for unfavorable economic conditions in affected populations (3–5).

Conditional cash transfer programs (CCTs) have been proposed as a promising, cost-effective strategy for overcoming intergenerational poverty and ameliorating the social determinants of health (6). However, there is limited evidence of their impact on neglected tropical diseases (7). The Brazilian CCT, the Bolsa Familia Program (BFP), provides financial aid to low-income families, conditional on school attendance and preventive health checkups, and has been linked to improvements in children’s education, health-care access, and food security (8–12). Although leprosy in Brazil has been declining in the past decades, Brazil still registers over 20,000 new leprosy cases annually, accounting for over 14% of cases diagnosed globally (13). Higher CCT coverage has been associated with reductions in leprosy risk at the population level (14, 15). However, no studies to date have provided a robust assessment of the impact of BFP or any CCTs on the burden of leprosy using individual-level data. (11)

To address this gap, we tested the hypothesis that receiving BFP can reduce leprosy incidence, using prospective data that was routinely collected from families enrolled in the Brazilian National Registry for Social Programs Cadastro Único (CadUnico), the BFP Payroll Database, and the Brazilian Notifiable Disease Registry (SINAN), and linked as part of the 100 Million Brazilian Cohort.

## METHODS

### Intervention

The BFP targets families registered in CadUnico who live in: 1) extreme poverty (i.e., earning ≤60 Brazilian Real (BRL) per capita/month in 2007–2008 and ≤70 BRL per capita/month in 2009–2014); or 2) poverty (i.e., ≤120 BRL per capita/month in 2007–2008 and ≤140 BRL per capita/month in 2009–2014) with ≥1 child (i.e., <18 years old) and/or with a woman who is pregnant or breastfeeding (Web Appendix 1, Web Tables 1–8, available at https://academic.oup.com/aje). One BRL = approximately 0.25 US dollars. The BFP provides monthly payments to families conditional on compliance with: 1) children’s attendance for ≥80% of school days; 2) health monitoring of children ≤6 years of age and breastfeeding women; and 3) prenatal care (see Web Appendix 1 for further details).

### Data sources and linkage

The 100 Million Brazilian Cohort is a large-scale linked cohort that aims to evaluate the impact of the BFP and other social programs on health outcomes in Brazil (16). For the current investigation, we linked the baseline of the 100 Million Brazilian Cohort, the BFP Payroll Database (2004–2015), and SINAN (2007–2014) (17). See Web Appendix 2 for linkage details.

The 100 Million Brazilian Cohort baseline covariates comprised those from the first registry of families in CadUnico: sociodemographic variables (i.e., sex, age, self-identified race/ethnicity, education, and work) for the head of family (i.e., oldest member), the state and area of residence (urban vs. rural), household living conditions (i.e., house ownership, housing material, water supply, electricity, sewage, and waste collection), per capita income, and individual-level identifiers for linkage (i.e., Social Identification Number (NIS), name, date of birth, sex, maternal name, and municipality). Exposure data extracted from the BFP Payroll Database included starting and end dates of BFP benefit receipt for each primary recipient per family and the individual-level identifier for linkage (i.e., Social Identification Number). Outcome data extracted from SINAN included: date of leprosy diagnosis, clinical presentation (i.e., paucibacillary (PB): ≤5 lesions; or multibacillary (MB): >5 lesions or positive slit skin smear), and disabilities at diagnosis (i.e., grade 0 if no disabilities or grade 1/2 with any sign of eye problems, visible deformity, damage, or anesthesia in hands and feet) (2), and individual-level identifiers for linkage (i.e., name, date of birth, sex, maternal name, and municipality).

The 100 Million Brazilian Cohort baseline and BFP data sets were deterministically linked using a unique identifier (i.e., Social Identification Number). The cohort baseline and SINAN data sets were linked by the 5 individual-level identifiers in 2 steps using the CIDACS-RL tool (https://gitHub.com/gcgbarbosa/cidacs-rl) (16). In the first step, entries were deterministically linked. In the second step, entries that were not linked deterministically were then linked based on a similarity score between all the pairwise comparisons (i.e., ranging from 0 to 1); entries with the highest similarity scores were considered to be linked pairs.

To assess the accuracy of the linkage procedures, we performed a manual validation study; 10,000 pairs were randomly selected from all possible paired links. Manual verification was used to classify pairs as true or false links. Various cutoffs of the similarity score were used to declare pairs to be a link. These linkages were compared with the true link status to determine the sensitivity and specificity at each potential cutoff. The cutoff corresponding to the optimal sensitivity and specificity was chosen (a similarity score of 0.92) to determine links for this study (Web Appendix 2) (18). Following linkage, individual identifiers were removed from the data set.

### Selection of the study population

**Figure 1:**
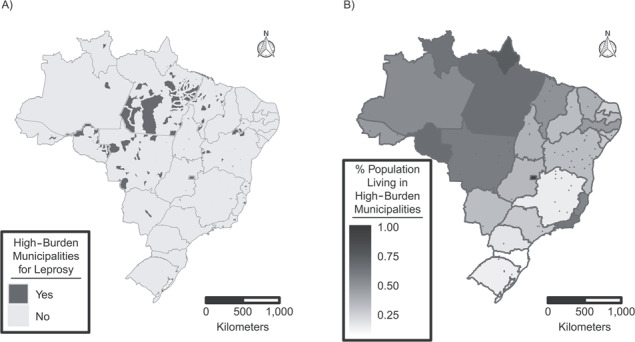
Map of Brazil, showing the priority municipalities for leprosy control in Brazil designated as high-burden (A) and the proportion of individuals residing in high-burden municipalities according to state (B) for the 26 Brazilian states and the Brazilian Federal District, 2016. High-burden municipalities include all state capitals, municipalities in high-risk areas for leprosy with a leprosy new-case detection rate ≥20/100,000 or ≥20 new cases or ≥10 new cases, with at least 1 case in children under age 15 years in 2010, and municipalities outside the geographical risk areas with ≥50 new cases, with at least 5 cases in children under 15 years of age in 2010.

The study population included individuals belonging to families who enrolled in the 100 Million Brazilian Cohort between January 2007 and December 2014. We excluded families who: 1) lacked at least 1 individual over 15 years of age at enrollment (i.e., children recorded separately from an adult caregiver were not considered to be a family); 2) had a monthly per capita income exceeding 5,000 BRL; and/or 3) started receiving BFP benefits prior to enrollment. We defined as BFP beneficiary families those that started receiving BFP benefits within 6 months after enrollment in the cohort (i.e., reflecting the typical time to receipt for families who would eventually become beneficiaries) and nonbeneficiary families (i.e., non-BFP) those that did not start receiving the benefit within 6 months after enrollment (Web Figure 1). We analyzed the overall sample and then stratified our study population by whether or not families resided in one of the 182 “priority” municipalities in Brazil as officially designated due to their high burden of leprosy (Figure 1) (19).

### Statistical analysis

#### Propensity score matching.

We used propensity score (PS) matching to compare BFP beneficiary (i.e., exposed) and nonbeneficiary (i.e., unexposed) families. We estimated the PS by multiple logistic regression using baseline sociodemographic characteristics and year of application for each data set (i.e., overall sample, high-burden municipalities, and low-burden municipalities) (Web Figure 1). Missingness in PS covariates was considered as a category. We performed 1:1 nearest-neighbor matching with a caliper of 0.05, allowing a same nonbeneficiary family to match with more than 1 beneficiary family (i.e., matching with replacement) (20). We compared the difference in the distribution of PS covariates between beneficiary and nonbeneficiary families using the standardized mean difference to assess balance of potential confounders before and after matching (standardized mean difference of >0.1 was taken to indicate potential confounding by that characteristic) (21). See Web Appendix 3 for matching details.

#### Primary and secondary outcome analysis.

Incident cases were defined as the first newly detected case of leprosy occurring within family units after enrollment. Secondary endpoints for leprosy incidence included operational classification (i.e., PB and MB) and the presence of disabilities at diagnosis (i.e., grade 0 and grade 1/2). Families with a leprosy case diagnosed prior to or within the first 6 months after enrollment were not considered disease-free at baseline and were therefore excluded from the analyses. For family units with more than 1 case occurring during the study period, only the first case was considered in the analysis. Family-years at risk began 6 months after enrollment (i.e., the time at which exposure status was determined) and ended on December 31, 2014, or at diagnosis of the first new leprosy case in the family. The total person-years at risk for each family was defined as the contribution of each family-year at risk multiplied by the number of individuals in the family. Unexposed families who later participated in BFP were censored at the time they started receiving BFP benefits. Given that the potential benefits of BFP participation (e.g., via behavior changes associated with the conditionalities) could persist after families stopped receiving the cash transfer benefit itself, BFP-exposed families remained in the exposed group during the full study period. For analyses of secondary endpoints, families were censored at the first new leprosy diagnosis if that diagnosis was an operational classification/grade of disabilities other than the one being considered or if it was missing.

We estimated the incidence rate ratios of leprosy new-case detection rate in the family (i.e., familial detection rate) in the matched cohort using Mantel-Haenszel (MH) tests and Poisson regressions with further adjustment for per capita income and robust standard errors clustered by family to account for matching with replacement. The person-years at risk were included in the model as an offset variable. We estimated the cumulative incidence rate ratio for beneficiary and nonbeneficiary families of BFP over time using the Nelson-Aalen estimator (22, 23). In addition, we estimated the association of BFP participation and familial detection rate of leprosy using Poisson regression models stratified by duration of follow-up (i.e., 0–6 months of exposure, 6 months and 1 day to 12 months, 1 year and 1 day to 2 years, 2 years and 1 day to 3 years, and >3 years).

**Figure 2:**
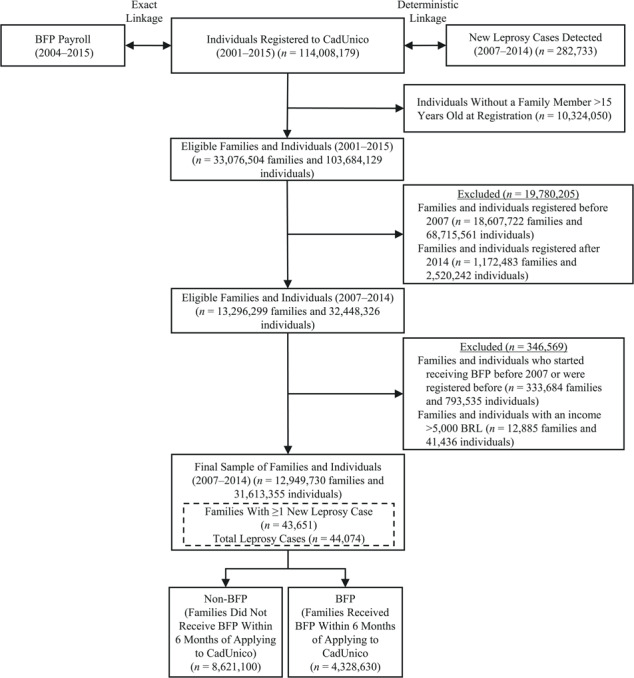
Flowchart of the study population, Brazil, 2001–2015. BRL, Brazilian reals; BFP, Bolsa Familia Program; CadUnico, Brazilian National Registry for Social Programs Cadastro Único.

**Figure 3:**
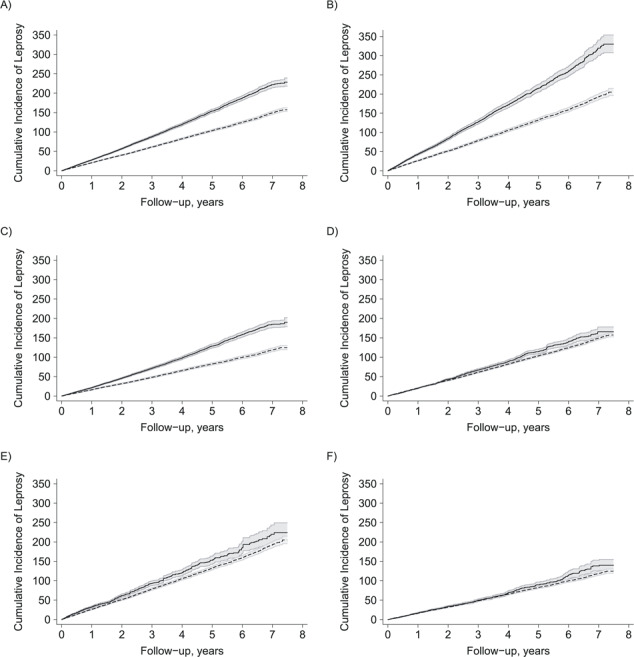
Cumulative incidence of leprosy among families (per 100,000) defined as receiving Bolsa Familia Program benefits within 6 months of enrollment in our cohort baseline (dashed line) and Bolsa Familia Program nonbeneficiary families (solid line) in the crude cohort, Brazil, 2007–2014. A) Overall; B) leprosy high-burden municipalities; C) leprosy low-burden municipalities. In the matched cohort: D) overall; E) leprosy high-burden municipalities; F) leprosy low-burden municipalities, according to follow-up time.

#### Sensitivity analyses.

To account for the possibility that some individuals might have started receiving BFP after the sixth month, we also analyzed BFP as a time-varying exposure. In this analysis, families that started receiving BFP between 6 months and 1 year after registration in CadUnico switched to the exposed group from 1 year on and were matched to families who had not received BFP by 1 year; similarly, families receiving by 1 year and 1 day to 2 years, and by 2 years and 1 day to 3 years, switched to the exposed group and were matched to families remaining unexposed (see Web Appendix 4, Web Figure 1). To explore the robustness of our results to the way income was accounted for, because this is an important factor due to being the main eligibility criterion for BFP, we excluded income from the Poisson regression model and adjusted for income using restricted cubic splines. Additionally, we estimated the association of BFP using inverse probability of treatment weighting and restricted the analysis to complete cases (i.e., excluding participants with missing data for any covariate in the PS model) (Web Appendix 5). To test whether there were potential biases due to differential loss of follow-up between BFP and non-BFP beneficiary families, we censored each matched pair by the smallest contribution of family-years at risk for them to contribute to the same number of family-years at risk. Finally, to test whether there was competing risk bias due to lack of mortality information in our cohort, we limited the follow-up time of each matched pair to 2 years.

All analyses were performed using STATA, version 15.0 (StataCorp LLC, College Station, Texas), and R, version 3.5.2 (R Foundation for Statistical Computing, Vienna, Austria) (packages: dplyr, brmap, descr, ggplot2, ggthemes, gridExtra, grid, readxl, reshape2, and ggfortify).

This study was performed under the international (Helsinki), Brazilian, and UK research regulations and was approved by the research ethics committee of 3 institutions: University of Brasília (1.822.125), Instituto Gonçalo Muniz-Fiocruz (1.612.302), and London School of Hygiene and Tropical Medicine (10580–1).

## RESULTS

Of the 37,285,406 individuals in the 100 Million Brazilian Cohort who registered to CadUnico between 2007 and 2014, 31,613,355 individuals from 12,949,730 families were investigated in this study (Figure 2). From this sample, we identified 44,074 new leprosy cases among families in the cohort baseline between 2007 and 2014. This represents 94% (44,074/46,856) of the number of cases expected if the cohort had similar leprosy incidence to the whole Brazilian population and 15.6% of the cases diagnosed in Brazil in the same period (Web Appendix 2, Web Table 1). After enrollment in the cohort, 4,328,630 commenced BFP participation within 6 months and an additional 2,865,583 of the included families started BFP benefits after that period. Among the 4,459,239 families living in high-burden leprosy municipalities, 41.9% (1,868,116/4,459,239) started benefiting from BFP within 6 months; among the 8,490,491 families living in low-burden municipalities, 29.0% (2,460,514/8,490,491) started receiving BFP benefits within the same period.

Overall, leprosy cases were detected in 43,651 families in the cohort, of which 22,301 occurred after enrollment. Over the study period, from 2007–2014, leprosy incidence rates remained constant in BFP beneficiary and non-BFP beneficiary families from our cohort (Web Appendix 6, Web Figure 2). Out of the 22,301 incident cases, 8,622 (38.7%) cases were classified as PB, 13,661 (61.3%) as MB, and 18 (0.1%) as missing data on operational classification. Of cases, 13,777 (61.8%) were diagnosed without disabilities, 6,290 (28.2%) were diagnosed with leprosy-associated disabilities (grade 1/2), and for 2,234 cases (10.0%) grade of disabilities was not recorded. Overall, the familial detection rate was 17.4/100,000 person-years at risk (95% confidence interval (CI): 17.1, 17.7) and substantially higher in “priority” (high-burden) compared with “nonpriority” (low-burden) municipalities (familial detection rate = 22.8/100,000, 95% CI: 22.2, 23.3, vs. familial detection rate = 14.3/100,000, 95% CI: 14.0, 14.7). Crude cumulative leprosy incidence among families was markedly lower among BFP than among non-BFP beneficiaries (crude relative risk (RR)_MH_ = 0.70, 95% CI: 0.68, 0.73), with similar differences in high-burden (crude RR_MH_ = 0.62, 95% CI: 0.59, 0.65) and in low-burden municipalities (crude RR_MH_ = 0.69, 95% CI: 0.66, 0.72) (Figure 3A–C).

At baseline, there were significant differences between families who received BFP benefits (hereafter, BFP) and those that did not (hereafter, non-BFP) (Table 1). Relative to non-BFP participants, BFP family heads were more likely to be female (60.7% vs. 53.4%) and were younger (median age 32.6 vs. 40.2 years). BFP families also had relatively higher median numbers of individuals per family (3 vs. 2), and lower median monthly per capita income (50.0 BRL vs. 177.7 BRL, equivalent to 6.9% and 24.5% of the 2014 minimum wage). PS matching successfully matched >99.9% of the BFP families with similar non-BFP families in all matched samples (See Web Appendix 3, Web Figures 3–4, and Web Tables 4–5 for details of the PS analysis).

After matching, using Mantel-Haenszel tests or Poisson regression models with further adjustment for income, BFP was not associated with lower familial detection rate of leprosy in our overall sample (incidence rate ratio (IRR)_MH_ = 0.96, 95% CI: 0.92, 1.00; IRR_Poisson_ = 0.97, 95% CI: 0.90, 1.04), but BFP beneficiary families living in high-burden municipalities had a substantially lower familial detection rate of leprosy (IRR_Poisson_ = 0.86, 95% CI: 0.77, 0.96) (Table 2). In high-burden municipalities, the point estimates for the association between BFP and leprosy was more extreme for the detection of leprosy-associated disabilities (IRR_Poisson_ = 0.79, 95% CI: 0.65, 0.97) and paucibacillary cases (IRR_Poisson_ = 0.82, 95% CI: 0.68, 0.98).

The cumulative familial detection rate of leprosy was initially similar between beneficiary and nonbeneficiary families (Figure 3C and 3D). However, after 2 years, the accrual of new cases detected was markedly lower among beneficiary families, and the difference in familial detection rate according to exposure status was larger among the families living in high-burden municipalities (Figure 3D). Also, by using Poisson models stratified temporally, the point estimate for the association between BFP receipt and leprosy familial detection rate indicated slightly higher detection in the first 6 months on benefits but lower familial detection rate thereafter (Web Table 5 and Web Figure 5). Similar trends were observed for secondary endpoints related to grade of disabilities, but no differences over time were observed according to operational classification (Web Table 5 and Web Figure 5).

In sensitivity analysis, when allowing treatment to vary over time, we obtained similar but less extreme point estimates for the association between BFP and leprosy primary endpoints (Web Table 6). We also obtained similar point estimates for the association between receiving BFP and familial detection rate of leprosy when using Poisson regression without further adjusting for income (IRR_Poisson_ =0.96, 95% CI: 0.89, 1.03) and when further adjusting for income using spline (IRR_Poisson_ = 0.93, 95% CI: 0.87, 1.00) (Web Table 7). Inverse probability of treatment weighting also generated similar estimates to the primary analysis, suggestive of slightly lower leprosy incidence among BFP beneficiary families (IRR_Poisson_ = 0.95, 95% CI: 0.90, 1.00) and stronger point estimates among cases with disabilities (IRR_Poisson_ = 0.87, 95% CI: 0.79, 0.96) (Web Table 8). The complete-case analysis included 2,695,543 (63%) of the original BFP beneficiary families and yielded results similar to the primary analysis (IRR_Poisson_ = 0.99, 95% CI: 0.90, 1.10) (Web Table 9). When considering the same number of family-years at risk for each matched pair or restricting the follow-up to 2 years, we also obtained similar or more extreme point estimates (Web Table 10 and Web Table 11).

**Table 1:** Description of Nonbeneficiary and Beneficiary Families of the Bolsa Familia Program (*n* = 12,949,730) Within 6 Months of Registration in the 100 Million Brazilian Cohort, Brazil, 2007–2014

**Social and Demographic Variable**	**Non-BFP (8,621,100)**			**BFP (4,328,630)**			
	**No.**	**%**	**Median (IQR)**	**No.**	**%**	**Median (IQR)**	***P* Value** ^**a**^
Head-of-family characteristics							
Age, years			40.2 (26.1–59.2)			32.6 (26.4–42.1)	<0.001
Sex							<0.001
Male	4,017,128	46.6		1,699,124	39.3		
Female	4,603,972	53.4		2,629,506	60.7		
Ethnicity							<0.001
White	2,910,212	33.8		1,222,972	28.3		
Black	625,762	7.3		389,069	9.0		
Asian	43,379	0.5		18,047	0.4		
Mixed/Brown	4,566,436	53.0		2,465,304	57.0		
Indigenous	19,571	0.2		51,036	1.2		
Missing	455,740	5.3		182,202	4.2		
Literacy							<0.001
Yes	7,364,320	85.4		3,875,678	89.5		
No	1,220,161	14.2		422,482	9.8		
Missing	36,619	0.4		30,470	0.7		
Education^**b**^							<0.001
Primary school or less	2,495,387	28.9		1,090,961	25.2		
Junior high school	2,069,347	24.0		1,387,715	32.1		
High school	2,055,301	23.8		990,945	22.9		
Missing	2,001,065	23.2		859,009	19.8		
Occupation							<0.001
Currently not working	3,720,000	43.1		1,995,758	46.1		
Working	3,996,326	46.4		1,773,206	41.0		
Missing	904,774	10.5		559,666	12.9		
Household characteristics							
Region of residence							<0.001
North	890,499	10.3		558,683	12.9		
Northeast	2,810,584	32.6		1,333,410	30.8		
Southeast	2,969,267	34.4		1,777,094	41.1		
South	1,112,028	12.9		357,311	8.3		
Midwest	838,722	9.7		302,132	7.0		
Area of residence							<0.001
Urban	7,055,818	81.8		3,548,224	82.0		
Rural	1,555,317	18.0		762,014	17.6		
Missing	9,965	0.1		18,392	0.4		
Leprosy high-burden municipality							
No	6,029,977	69.9		2,460,514	56.8		
Yes	2,591,123	30.1		1,868,116	43.2		
Type of household							<0.001
Private	7,435,902	86.3		3,630,445	83.9		
Shared and informal housing	342,213	4.0		174,306	4.0		
Missing	842,985	9.8		523,879	12.1		
Construction material							<0.001
Bricks/cement	6,894,374	80.0		3,402,241	78.6		
Wood, other vegetal materials	1,436,989	16.7		794,292	18.3		
Missing	289,737	3.4		132,097	3.1		
Water supply							<0.001
Public network (tap water)	6,541,878	75.9		3,120,429	72.1		
Well, natural sources or other	1,789,487	20.8		1,076,099	24.9		
Missing	289,735	3.4		132,102	3.1		
Electricity							<0.001
Electricity with counter	7,579,449	87.9		3,528,588	81.5		
Electricity without counter or no electricity	751,916	8.7		667,939	15.4		
Missing	289,735	3.4		132,103	3.1		
Sewage							<0.001
Public network or septic tank	5,599,038	64.9		2,769,506	64.0		
Homemade septic tank, ditch, or other	2,533,943	29.4		1,305,835	30.2		
Missing	488,119	5.7		253,289	5.9		
Waste							<0.001
Public collection system	6,898,397	80.0		3,430,663	79.3		
Burned, buried, outdoor disposal, or other	1,432,970	16.6		765,868	17.7		
Missing	289,733	3.4		132,099	3.1		
Basic services^**c**^							<0.001
All adequate	4,669,921	54.2		2,146,962	49.6		
1 inadequate	1,832,776	21.3		935,173	21.6		
2 or 3 inadequate	799,949	9.3		486,692	11.2		
All inadequate	830,326	9.6		506,507	11.7		
Missing (all)	488,128	5.7		253,296	5.9		
Family members			2 (1–3)			3 (2–4)	<0.001
Residents per room			0.5 (0.3–0.8)			0.8 (0.5–1.0)	<0.001
No. of children <18 years old			0 (0–1)			1 (1–2)	<0.001
No. of elders >60 years old			0 (0–0)			0 (0–0)	<0.001
Family income, BRL			465 (110–724)			150 (30.0–300.0)	<0.001
Per capita income, BRL			177.7 (50–428.449)			50.0 (11.4–90.0)	<0.001

Abbreviations: BFP, Bolsa Familia Program; BRL, Brazilian real; IQR, interquartile range.

^a^ Two-tailed *t* test used for comparison of continuous variables and Pearson χ^2^ for categorical variables; missing data were considered a category.

^b^ Primary school or less: ≤5 years of education; junior high school: 6–9 years of education; high school: ≥10 years of education.

^c^ Basic services: water supply, electricity, sewage, and waste.

**Table 2:** Incidence Rate Ratio of Leprosy, Overall and According to Grade of Disabilities and Operational Classification, for the Bolsa Familia Program, Brazil, 2007–2014

**Case Variable** ^**a**^	**No. of Cases**	**IR in BFP**	**95% CI**	**IR in Non-BFP**	**95% CI**	**IRR**_**MH**_ ^**b**^	**95% CI**	**IRR**_**Poisson**_ ^**c**^	**95% CI**
Brazil overall									
All new cases^d^	9,886	14.84	14.50, 15.18	15.48	14.91, 16.07	0.96	0.92, 1.00	0.97	0.90, 1.04
Grade 0	6,371	9.65	9.38, 9.93	9.73	9.28, 10.20	0.99	0.94, 1.05	1.00	0.92, 1.10
Grade 1 or 2 disabilities	2,534	3.74	3.57, 3.92	4.13	3.84, 4.45	0.91	0.83, 0.99	0.92	0.80, 1.05
Paucibacillary cases	4,022	6.09	5.87, 6.31	6.15	5.79, 6.52	0.99	0.92, 1.06	0.99	0.89, 1.10
Multibacillary cases	5,860	8.74	8.48, 9.00	9.33	8.89, 9.79	0.94	0.88, 0.99	0.96	0.87, 1.05
Leprosy high-burden municipalities^e^									
All new cases^f^	5,394	18.97	18.38, 19.58	22.26	21.17, 23.40	0.85	0.80, 0.90	0.86	0.77, 0.96
Grade 0	3,620	12.99	12.50, 13.49	14.19	13.33, 15.11	0.92	0.85, 0.98	0.91	0.80, 1.04
Grade 1 or 2 disabilities	1,251	4.29	4.01, 4.58	5.50	4.97, 6.08	0.78	0.69, 0.88	0.79	0.65, 0.97
Paucibacillary cases	2,415	8.43	8.04, 8.84	10.16	9.44, 10.94	0.82	0.76, 0.91	0.82	0.68, 0.98
Multibacillary cases	2,978	10.54	10.10, 11.00	12.09	11.29, 12.94	0.87	0.81, 0.94	0.89	0.77, 1.02
Leprosy low-burden municipalities^g^									
All new cases^h^	4,578	11.82	11.43, 12.76	12.08	11.43, 12.76	0.98	0.92, 1.05	0.99	0.90, 1.09
Grade 0	2,746	7.22	6.92, 7.55	6.90	6.41, 7.42	1.05	0.96, 1.14	1.06	0.94, 1.20
Grade 1 or 2 disabilities	1,319	3.35	3.14, 3.57	3.64	3.30, 4.03	0.92	0.82, 1.03	0.93	0.79, 1.11
Paucibacillary cases	1,672	4.39	4.15, 4.64	4.22	3.85, 4.63	1.04	0.93, 1.16	1.04	0.89, 1.21
Multibacillary cases	2,903	7.42	7.11, 7.75	7.85	7.34, 8.41	0.95	0.87, 1.02	0.96	0.86, 1.09

Abbreviations: BFP, Bolsa Familia Program; CI, confidence interval; IR, incidence rate; IRR, incidence rate ratio; MH, Mantel-Haenszel.

^a^ For *n* = 8,545,694 families; family-years at risk = 23,467,162.1; person-years at risk = 65,878,418.7.

^b^ IRR estimated using Mantel-Haenszel method.

^c^ IRR estimated using Poisson regression adjusting for income (continuous) and including robust standard errors clustered by family.

^d^ In the stratified analysis, cases missing grade of disabilities at diagnosis (*n* = 981) or operational classification (*n* = 4) were censored at the time the leprosy case occurred.

^e^ For *n* = 3,674,130 families; family-years at risk = 9,707,927; person-years at risk = 27,235,798.9.

^f^ In the stratified analysis, cases missing grade of disabilities at diagnosis (*n* = 523) or operational classification (*n* = 1) were censored at the time the leprosy case occurred.

^g^ For *n* = 4,871,424 families; family-years at risk = 13,719,482.8; person-years at risk = 38,493,252.5.

^h^ In the stratified analysis, missing in grade of disabilities at diagnosis (*n* = 513) or operational classification (*n* = 3) were censored at the time the leprosy case occurred.

## DISCUSSION

This study investigated the impact of the Brazilian CCT program on new-case detection of leprosy in a subset of the 100 Million Brazilian Cohort, which included 31.6 million individuals from over 12.9 million families. Our findings suggested that BFP was associated with lower incidence of leprosy among families living in high-burden municipalities for the disease in Brazil. We also obtained stronger point estimates for the association between BFP and lower incidence of paucibacillary forms and leprosy-associated disabilities. These findings underscore the potential value of CCTs for the control of leprosy in low- and middle-income countries.

Our results indicated that families enrolled in the BFP between 2007 and 2014 who resided in high-burden municipalities for leprosy had a 14% lower leprosy familial detection rate relative to nonbeneficiary families. These results point to a similar magnitude of the association between BFP and leprosy risk to that previously described in ecological studies (14, 24). These ecological studies have reported a 15% lower leprosy new-case detection rate in the general population and in children under 15 years of age in Brazilian municipalities with high BFP coverage (≥48% coverage) compared with municipalities with low coverage (<28%) (14, 24). Additionally, our study suggests that BFP is associated with fewer leprosy cases with PB presentations and among cases with disabilities, although point estimates were consistent across the other clinical presentations (i.e., MB forms and cases without disabilities). Due to the importance of reducing leprosy and related disabilities, these findings are of particular relevance to leprosy-control strategies (2, 25).

CCTs are designed to have both short- and long-term impacts on beneficiary families (26). By following families for up to 7.5 years, our study provides new evidence that the association between BFP and lower leprosy incidence was more prominent after a minimum of 2 years in the program. The delayed association of the BFP could be partially explained by the chronic nature of leprosy, which has an incubation period of up to 10 years (27). It is plausible that in the short term, BFP could increase food availability and bolster host immunity, while cumulative exposure to BFP could influence leprosy risk through longer-term mediators, such as education, crowding, and other social determinants of health (4, 11, 28). Stronger point estimates for the association between BFP and incidence of PB leprosy forms and leprosy-associated disabilities in high-burden leprosy municipalities deserve further consideration. Because leprosy-associated disabilities can be prevented by early detection, enhanced health-care utilization rates among beneficiary families could mediate the observed lower incidence of cases with disabilities (11). Nevertheless, there is poor knowledge of the factors that mediate different immune response in PB and MB leprosy that could explain why receiving BFP is associated with more pronounced association with lower PB leprosy forms in comparison with MB forms. Also, increased access to health care among beneficiary families might increase leprosy detection, and it is therefore likely that our results represent an underestimate of the causal effect of BFP on leprosy incidence in Brazil. Although further research on the underlying mechanism by which BFP affects leprosy risk in high-burden municipalities is warranted, our results indicate that CCTs might have the greatest impact in scenarios where individuals face a higher and less heterogeneous disease risk (29).

The strengths and limitations of this study warrant consideration. The 100 Million Brazilian Cohort is a powerful resource of sociodemographic information covering the poorest half of the Brazilian population. Although previous studies have evaluated the association between BFP and leprosy or tuberculosis incidence in Brazil at the ecological level (14, 15), this is the first study to use linked administrative data to study the potential impact of a nationwide cash transfer program on infectious disease incidence at the individual level. Further, because leprosy is a rare disease, the large size of the analytical cohort provided unprecedented power to evaluate the associations between BFP and leprosy, as well as its understudied clinical manifestations. Finally, our analysis remained consistent, with similar point estimates in all sensitivity analyses conducted, including inverse probability of treatment weighting and restricted follow-up times. However, our study is also subject to limitations. First, although SINAN has national coverage, selection bias might have arisen due to the suboptimal linkage between the leprosy registry and the cohort baseline. This might be explained by potential heterogeneity in the quality of leprosy notification across Brazil, given that individuals of mixed ethnicity and those living in the North and Northeast regions of the country appeared to be underrepresented among linked leprosy cases. Second, by defining exposure status at 6 months, we might have missed a very short-term impact of BPF participation in increasing leprosy diagnosis. Finally, residual confounding (e.g., distance to health clinics and/or access to primary health care) remains a concern even though key sociodemographic risk markers for leprosy were included in our propensity scores (4). Because this is a quasiexperiment, we are cautious regarding our causal claims. Nevertheless, given that leprosy prevalence is low and the incubation period is long, as well as the nature of BFP as a nationwide social intervention, it would be very unrealistic to conceive a randomized control trial in this context.

This study has shown that the low-cost BFP (i.e., costing <0.4% of the Brazilian GDP in 2007) is associated with a significant reduction of leprosy in high-burden settings, including cases with grade-2 disabilities that are the focus of the WHO Global Leprosy Strategy 2016–2020 (2). We hypothesize that CCTs might reduce infectious disease morbidity, in part, by addressing some of the underlying determinants of health, such as poverty, education, health-care access, and nutrition (5, 7, 28, 30, 31). A hundred years ago, it was stated that leprosy can be controlled with social development (32). Now we have scientifically demonstrated that social policies, such as BFP, could be a pillar for leprosy control, and perhaps contribute to its elimination. Although BFP has nationwide coverage, further efforts should be made to scale the program up to serve poor families that are just above the program eligibility threshold and living in municipalities with high leprosy risk. In conclusion, these findings indicate that relatively small cash transfer payments undertaken as part of long-term investment in social policies might have an important role in the control of a poverty-driven disease like leprosy.

## Supplementary Material

Web_Material_kwaa127
