# Supplementary material for: Conditional Cash Transfer Program and Leprosy Incidence: Analysis of 12.9 Million Families From the 100 Million Brazilian Cohort
Source: Am J Epidemiol. 2020 Jul 8;189(12):1547–58. doi: 10.1093/aje/kwaa127 (PMC7705605; doi:10.1093/aje/kwaa127)
Supplement: Web_Material_kwaa127 [file web_material_kwaa127.pdf]

Web Material

Conditional Cash Transfer Program and Leprosy Incidence: Analysis of 12.9 Million Families from The 100 Million Brazilian Cohort

Julia M. Pescarini, Elizabeth Williamson, Maria Y. Ichihara, Rosemeire L. Fiaccone, Laura Forastiere, Anna Ramond, Joilda Silva Nery, Maria Lucia F. Penna, Agostino Strina, Sandra Reis, Liam Smeeth, Laura C. Rodrigues, Elizabeth B. Brickley**, Gerson O. Penna**, and Mauricio L. Barreto**.

** Joint senior authors

Table of contents

Web Appendix 1 2
Web Appendix 2 4
Web Appendix 3 5
Web Appendix 4 6
Web Appendix 5 8
Web Appendix 6 8
Web Figure 1 9
Web Figure 2 10
Web Figure 3 11
Web Figure 4 12
Web Figure 5 13
Web Table 1 15
Web Table 2 16
Web Table 3 17
Web Table 4 19
Web Table 5 22
Web Table 6 23
Web Table 7 24
Web Table 8 25
Web Table 9 25
Web Table 10 26
Web Table 11 26
References 27

Web Appendix 1

DATASETS – STRUCTURE AND IMPORTANT DEFINITIONS

The 100 Million Brazilian Cohort

The 100 Million Brazilian Cohort baseline is an open cohort using data linkage built by the Centre of Data and Knowledge Integration for Health (CIDACS/FIOCRUZ) [1,2]. The cohort is based on the idea of a "cohort baseline" with information of over 114 million individuals that were registered during 2001 and 2015 in the Brazilian National Registry for Social Programs – Cadastro Unico (CadUnico), which can be exposed to a certain social interventions and can be assessed on different health outcomes.
For the purpose of this study, we considered the intervention as the Bolsa Familia Program (BFP) and our study outcome was new cases of leprosy registered in the Brazilian Notifiable Disease Registry (SINAN). The baseline of the cohort that was produced by Cidacs/Fiocruz (Version 1) and linked with SINAN was available for the researches in January 2018 [3].

Brazilian National Registry for Social Programs – Cadastro Unico (CadUnico)

<u>Description</u>
CadUnico is a national administrative system containing information on all individuals, and their families, applying for any social programs in Brazil [4]. The registry system was established in 2001 aiming to integrate information from different cash transfer programs benefitting poor families in Brazil, such as gas aid, food, education, and child labor protection programs (i.e., *Auxílio-Gás*, *Bolsa Alimentação*, *Bolsa Escola*, and *Programa de Erradicação do Trabalho Infantil*). The registry was strengthened as an instrument for registering low-income families in Brazil after 2003, as a result of combining the former programs into a conditional cash transfer program, the BFP. In 2011, a new version of the CadUnico was implemented providing a better characterization of low-income families.

<u>Eligibility</u>
To be eligible for registration in CadUnico, families must earn a per capita income of up to half the minimum wage or a total familial income of up to 3 times the minimum wage (e.g., monthly minimum wage ranged from 380 BRL in 2007 to 724 BRL in 2014). By the end of 2015, CadUnico contained approximately 114 million individual registrations, representing just over 50% of the Brazilian population [4]. At registration, enrollees are assigned a unique social number identifier (NIS) and surveyed regarding socioeconomic indicators. Families should update their registry every 2 years while they remain enrolled in any social program. As the income threshold for BFP eligibility increased by 1.16 on August 2009, for this study, the baseline per capita income was divided by 1.16 for families registering with CadUnico after August 2009.

Bolsa Familia program

Description

BFP was created in 2003 to alleviate poverty and improve education and health [5]. Its implementation, in 2004, was followed by a formal analysis by the Brazilian government to define the BFP budget for each municipality in Brazil by estimating the number of individuals living in poverty and extreme poverty using the National Household Sample Survey (PNAD) [5]. Extremely poor families were considered to be those living with under 60 BRL per capita a month in 2007 and poor families were those living with under 120 BRL per capita a month in the same year, with frequent adjustments over the following years (Table S1).

Eligibility and cash payments

Enrollment to BFP is conditional on the family being registered in CadUnico and being eligible for the program (i.e., families living in extreme poverty or poverty). Each municipality was responsible for implementing the program and, due to administrative delay, eligible individuals could start receiving benefits at any time after application in CadUnico and consequently, in the 100 Million Brazilian Cohort baseline [2].

BFP benefit is given to a primary recipient, who should preferentially be a woman. Families considered as extremely poor receive a fixed sum plus a supplementary benefit for each applicable family member (i.e., children or adolescents up to 18 years old and women who are pregnant or breastfeeding) with a maximum of five supplementary benefits allocated per family. Families classified as poor receive only the supplementary benefits for applicable family members (Table S1). In 2011, the benefit system was extended to a maximum of seven supplementary benefits per family, and an extra supplement was provided to families who after allocation of BFP benefits remained classified as extremely poor, in order to allow these families to overcome the extreme poverty threshold [5]. Receipt of BFP benefits is subject to compliance with certain conditions: children or adolescents must attend school for at least 80% of school days, both children aged 0-6 and breastfeeding women must be monitored by health professionals, and pregnant women must receive prenatal care. Families receiving BFP benefits are monitored by social assistants to ensure that they maintain compliance with these conditions. In addition, families whose financial situation improves sufficiently, hence no longer meeting BFP poverty eligibility criteria, will continue receiving benefits for up to 2 years after they cease being eligible.

Brazilian Notifiable Disease Registry (SINAN)

Description

SINAN was first created in 1993 but only regulated in 1998, becoming the official Brazilian information system for over 40 notifiable health conditions, including leprosy[6]. Following detection of a leprosy case, health professionals, in both private and public services, are required to notify the electronic system, reporting date of detection and clinical and sociodemographic characteristics of the patient and update information regarding the treatment[6].

Web Appendix 2

DATA LINKAGE QUALITY AND BIAS ASSESSMENT

Methods for and bias assessment

Succeeding linkage between the baseline of the 100 Million Brazilian Cohort and SINAN-leprosy using the CIDACS-RL linkage tool [7], all registries from the baseline cohort received a similarity score (ranging from 0-1) attributed to a possible link with SINAN-leprosy. After that, we randomly select 10,000 linked pairs, and two independent researchers classified the pairs as true or false matches. Linkage accuracy was evaluated by calculating the sensitivity and specificity for various cut-offs of similarity score[7]. For each strata of similarity score, we were able to estimate the true positives, the false positives, the number of missed links (false negatives) and classified the remaining registries as non-links.

In addition, to evaluating potential bias due to the linkage process, we assessed whether the incidence of leprosy cases in the baseline of the 100 Million Brazilian Cohort was representative of the expected leprosy incidence in the total study population and by sociodemographic categories. We used sociodemographic characteristics available from the 2010 Brazilian population census which were also available in the cohort baseline and SINAN-leprosy: sex, age, ethnicity, region of residence and living in rural versus urban areas. We estimated the mean new case detection rate (NCDR) in the Brazilian population during the study period, as the ratio of yearly mean new leprosy cases from 2007-2014 obtained from SINAN-leprosy, over the total Brazilian population in 2010[8]. The NCDR estimated from the total population was used to calculate the expected number of cases occurring among the individuals registered in the baseline of the 100 Million Brazilian Cohort from 2007-2014, which we compared to the observed number of new leprosy cases linked.

Accuracy of the linkage using Cidacs-RL

Using the CIDACS_RL tool, linkage between the 100 Million Brazilian Cohort (2001-2015) and SINAN-leprosy (2007-2015) linked 54% of cases with a PPV of 90.6%, specificity of 0.89 (95%CI=0.88-0.90) and a sensitivity of 0.91 (95%CI=0.90-0.92) for the chosen cut-off point of 0.92. Comparing the expected and the observed number of new cases linked with our study population, linkage between individuals whose families were registered in the baseline of the 100 Million Brazilian Cohort from 2007-2014 with individuals diagnosed with leprosy in SINAN in the same period resulted in 44,074 new leprosy cases within the study population of 31,613,355. This represents 94% (44,074/46,856) of the expected number of cases linked given the size of the baseline of the 100 Million Brazilian Cohort from 2007-2014 used in this study or 15.6% of total new leprosy cases diagnosed in Brazil between 2007 and 2014 in Brazil (Table S2). There were no major differences in proportion of expected and observed linked cases overall, by sex or area of residence. Nevertheless, we observed that 15% fewer cases were linked among individuals with brown/mixed ethnicity, 7% fewer cases were linked in the Northeast, and 6% fewer cases were linked in the North region of Brazil. We also observed more cases linked among individuals over 25.

Web Appendix 3

PROPENSITY SCORE MATCHING

Definition of exposure groups

If eligible, families can start receiving BFP in any point in time after enrolment in cohort, but the majority of BFP beneficiaries start receiving BFP benefit within 6 months. Therefore, we defined exposure to BFP depending on whether households received benefits within 6 months after registration to the cohort. Therefore, families contributed to the analyses only as beneficiary or as non-beneficiary families.

We defined three datasets comprising: i) the overall sample, ii) households living in high burden municipalities for leprosy, and iii) households not living in priority municipalities for leprosy. In each of the three datasets, we excluded households that applied after July 2014 and stayed in our cohort for less than 6 months, and households where the first new leprosy case occurred before application to the cohort or during the first 6 months (see Figure S2).

Estimating logistic regression for each groups of exposure

For each dataset, we estimated the probability of receiving BFP benefit given the baseline covariates using multiple logistic regression (Table S3). Covariates included sex, age, ethnicity, education level and work of the head of the household (oldest family member), region and area of residence, house ownership, housing material, indicators of basic sanitation services (i.e., water supply, electricity, sewage and waste collection), per capita income in quintiles and year of application in the cohort baseline. Propensity score distribution for each dataset is shown in Figure S3.

Matching and post-estimation

Using 1:1 nearest-neighboring matching with a caliper of 0.05, we were able to match: all but 102 beneficiary families within 6 months in the overall sample, all but 19 beneficiary families living in high-burden municipalities for leprosy, and all but 153 families living in non-high-burden municipalities for leprosy (Figure S2). In the overall Brazilian sample, 0.6% (24,437/4,272,847) of the non-BFP families were matched 10 or more times with BFP families; 1% (18,744/1,837,065) of the non-BFP families living in high-burden leprosy municipalities; and 0.3% (7,090/2,435,712) of the non-BFP families living in non-priority municipalities for leprosy control were matched 10 or more times with BFP families living in the same type of municipalities.

Table S4 shows the distribution and the estimated standardized mean difference (SMD) across matching covariates between families exposed and non-exposed to BFP benefit in each of the three datasets after matching (Table S4). We also show, for the overall Brazilian matched cohort, the SMD before and after matching (Figure S4).

Web Appendix 4

TIME VARYING TREATMENT VARIABLE

Definition of exposure groups

To account for the fact that some families had a delay to over 3 years between application to the cohort and first receipt of BFP benefits, we defined exposure to vary over time. To account for that in the matching procedures, in addition to defining BFP families as those that start or did not start receiving BFP within 6 months, we also performed 3 additional analysis in order to, in each of them, include families exposed and families not-exposed to BFP in each given amount of time after applying to the cohort. In the first analysis, we defined as BFP beneficiary families those that start receiving BFP between the sixth month and 1 year of registration. In the second analysis, we defined as BFP beneficiary families those that start receiving BFP between 1 and 2 years of registration. In the third analysis, we defined as BFP beneficiary families those that start receiving BFP between 2 and 3 years of registration. Families that started receiving BFP after 3 years of registration were considered to be non-beneficiary families.

In each of the three additional analysis, we excluded (i) BFP beneficiary families already included in the previous analysis (e.g., we excluded families that were already defined as exposed to BFP within 6 months in the analysis considering BFP between 0.5 and 1 year after registration). Also, as in the primary analysis, we excluded; (ii) families where the first new leprosy case within the family unit occurred during the period of BFP definitions (e.g., for those families defined as beneficiaries or non-beneficiaries within six months and 1 year after applying to the cohort, we excluded all leprosy cases that occurred within the same period).

Estimating logistic regression for each groups of exposure, matching and post-estimation

For each of the exposed groups (i.e., three additional datasets), we estimated the probability of receiving BFP benefit given the same baseline covariates of the primary analysis using multiple logistic regression. To perform the matching, we used 1:1 nearest-neighboring matching with a caliper of 0·05. The datasets were matched separately and combined to the primary analysis producing a single/full matched cohort. Using this approach, families could be matched first as unexposed families and later as exposed, but never twice in the exposed groups. As in the primary analysis, after matching, we estimated leprosy incidence rate ratio between BFP and non-BFP families both using Mantel-Haenszel method and by using Poisson regression further adjusting for income and accounting for cluster robust standard errors by family. We performed the analysis for all families and separately for those living in high and low-burden leprosy municipalities.

Follow-up time calculation

As previously described for the primary analysis, for each matched cohort, the contribution of person-years at risk for each family began at the point where BFP exposure was defined (i.e., at 6 months, 1 year, 2 years and 3 years after registration in the cohort) and ended on December 31st 2014 or at diagnosis of the first new leprosy case in the family. Additionally, for unexposed families who later became exposed, the contribution of person-years for that family ended at the time of first receiving BFP benefits.

Results

The majority of families (4,328,630 families) received BFP within 6 months of applying to our cohort (primary analysis), 1,030,528 families received BFP within 6 months to 1 year (Group 2), 1,090,132 families received BFP between 1 to 2 years after applying (Group 3), and 453,930 received BFP between 2 to 3 years of applying to the cohort (Group 4). After combining the three groups with the primary analysis, our analysis included 13,506,522 matched families and yielded similar results to the primary analysis (Table S6).

Web Appendix 5

INVERSE PROPABILITY OF THE TREATMENT WEIGHTING (IPTW)

We used the same framework of analysis of the propensity score matching to estimate the effect of the treatment on the treated (ATT) using weights. First, we estimated the propensity score (ps) of receiving the BFP given the sociodemographic covariates of the cohort baseline for the overall sample and separately for each group of families living in high-burden or non-high burden municipalities for leprosy. Second, we estimated the weights for BFP beneficiary families (weight=1) and for non-BFP beneficiary families (weight= E(ps)/(1-E(ps)).

We estimated the incidence rate ratios (IRRs) of new case detection of leprosy for BFP and non-BFP beneficiary families for the overall sample (Table S6). The IRR was estimated using Poisson regression using inverse probability of treatment weighting (IPTW) with further adjustment for income. We investigated the dose-response effect of BFP participation on leprosy using analyses stratified by duration of exposure to BFP benefits (i.e., 0-6 months of fyr, 6-12 months, 1-2 years, 2-3 years, and 3+ years). Similarly to the main analyses using propensity score matching, we also investigated the association of BFP participation with the secondary outcomes of operational classification (i.e., PB versus MB) and presence of disabilities at diagnosis (i.e., G0D versus G1D/G2D).

Web Appendix 6

LEPROSY TRENDS AMONG BENEFICIARIES AND NON-BENEFICIARIES

We estimated leprosy trends in our dynamic cohort during 1st January 2007 to 31st December 2014 per semester of application into the study cohort. To do that, for each semester, we calculated the number of leprosy cases detected in the specific semester divided by the cumulative number of individuals among applicant families. The NCDR per 100,000 individuals per semester was calculated for BFP beneficiary and non-beneficiary families within 6 months after application to the cohort (Web Figure S2).

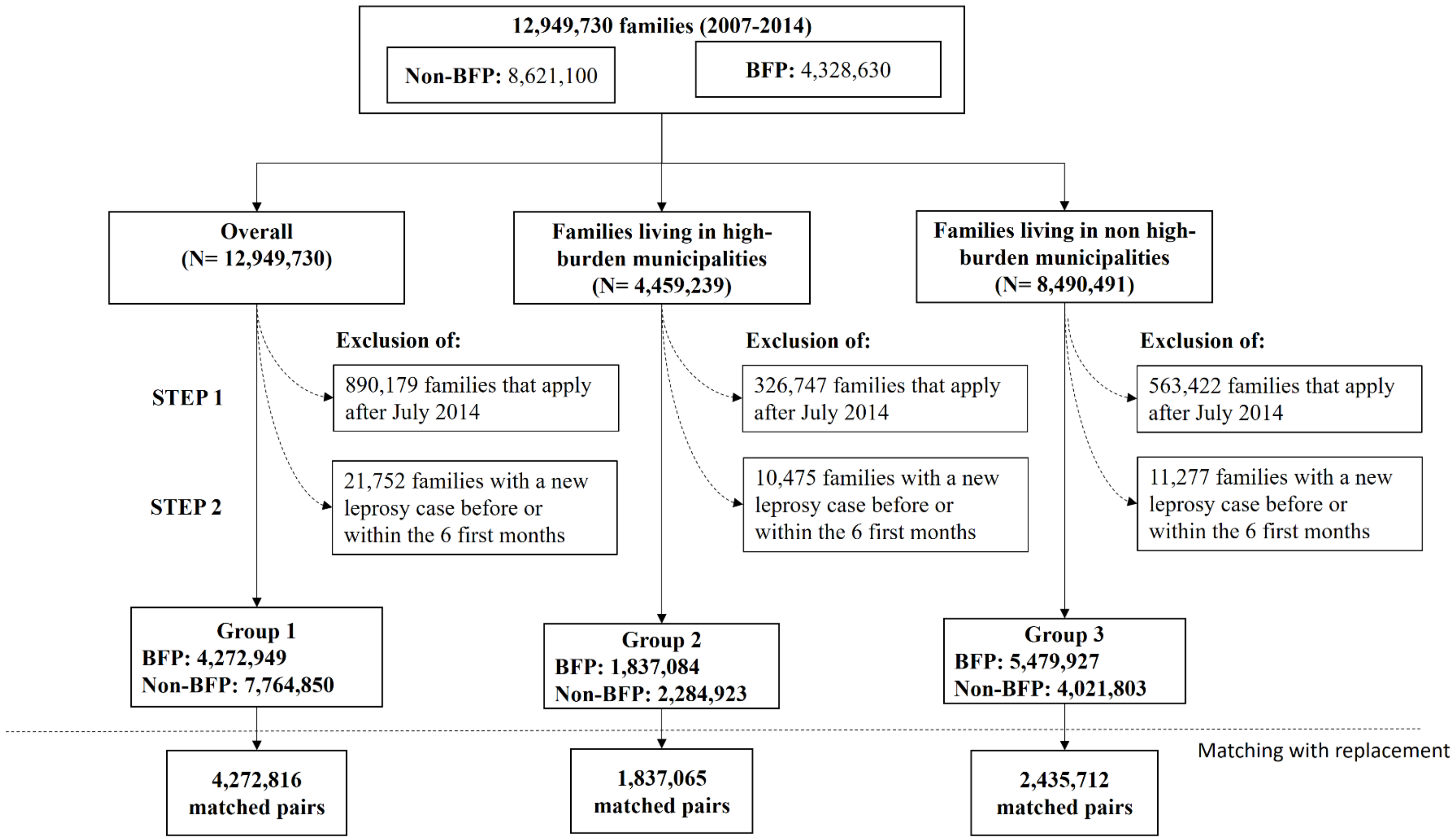

Web Figure 1 Description of the matching process in the overall sample and stratified in groups according to residence in municipalities with high leprosy burden.

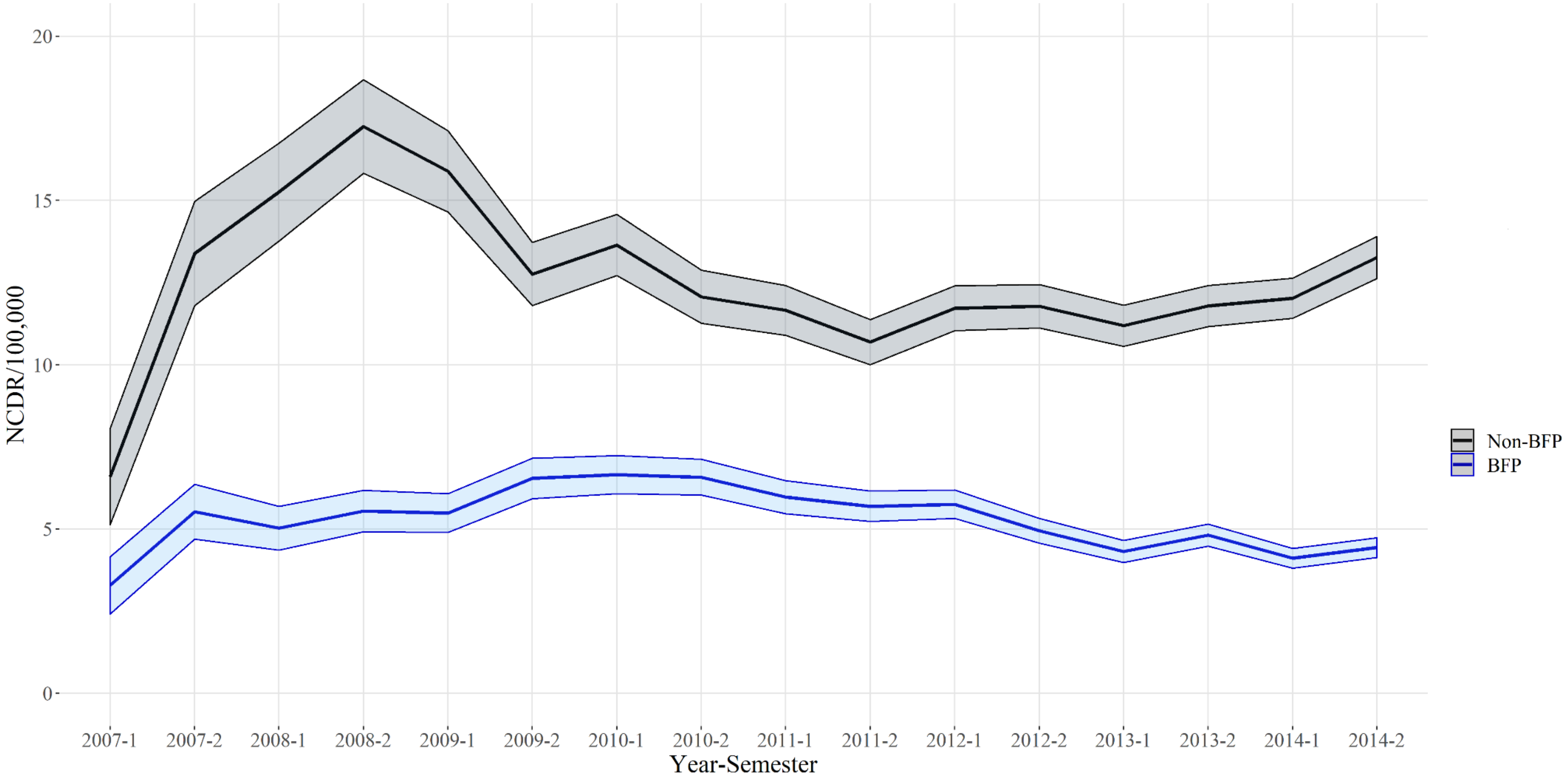

Web Figure 2 Incidence of leprosy by semester among individuals from BFP beneficiary families (blue) and non-beneficiary families (red) within 6 months of application to the cohort.

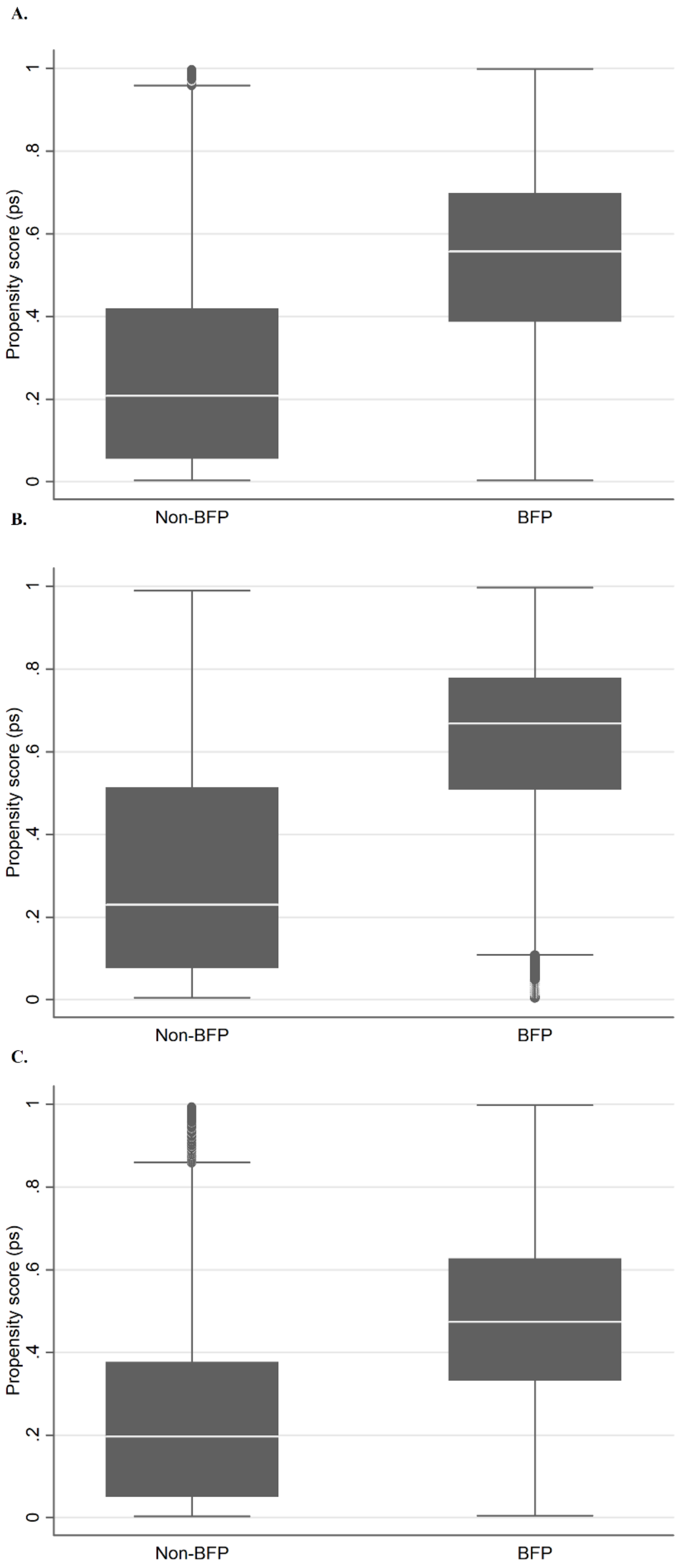

Web Figure 3 Distribution of propensity score in the overall sample (A), in high-burden municipalities for leprosy (B) and in non high-brden municipalities (C).

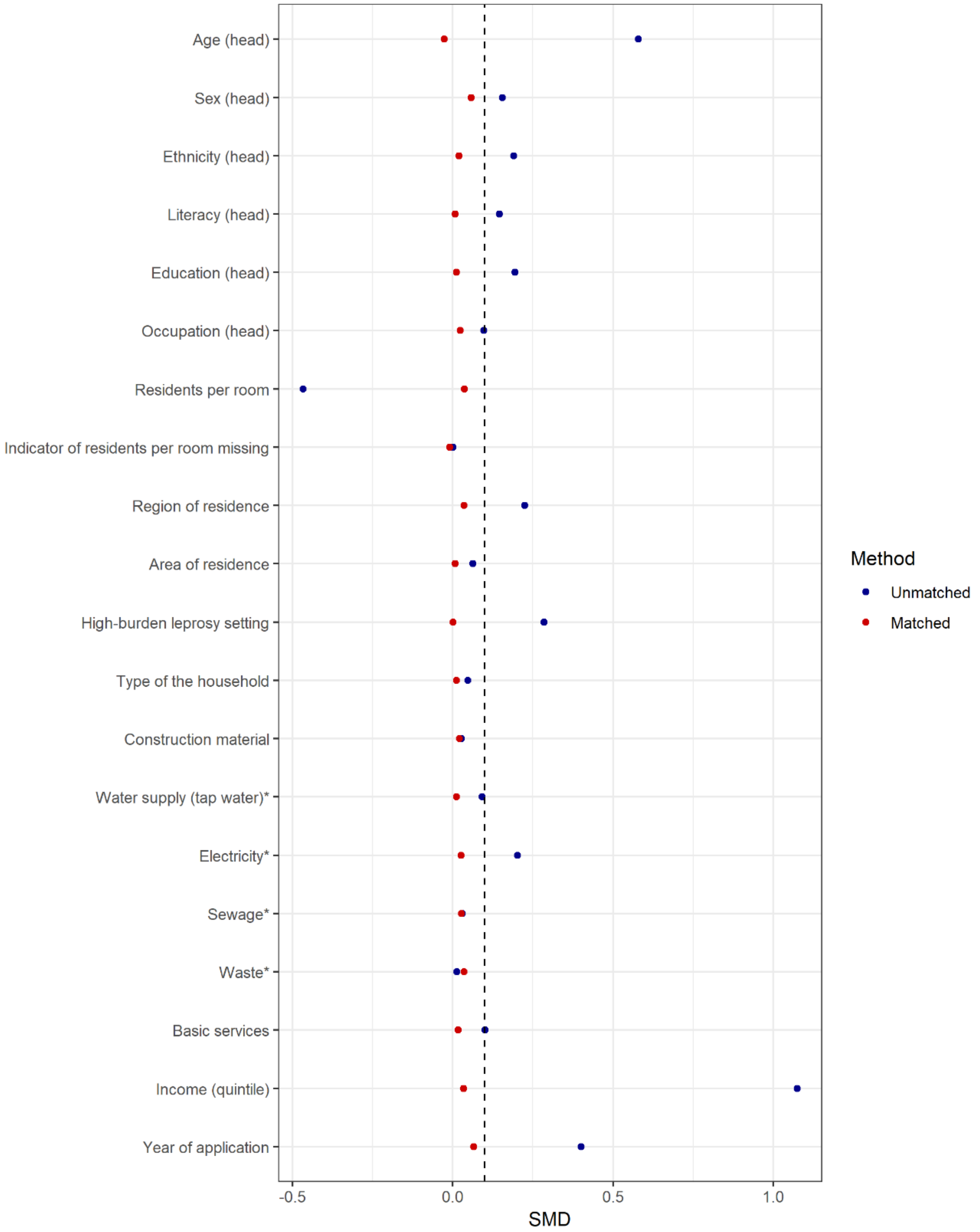

Web Figure 4 SMD between beneficiaries and non-beneficiaries of BFP for all the covariates before and after matching in the single matched dataset. SMD=Standardized mean differences; The black vertical dash line indicates SMD=0.1.

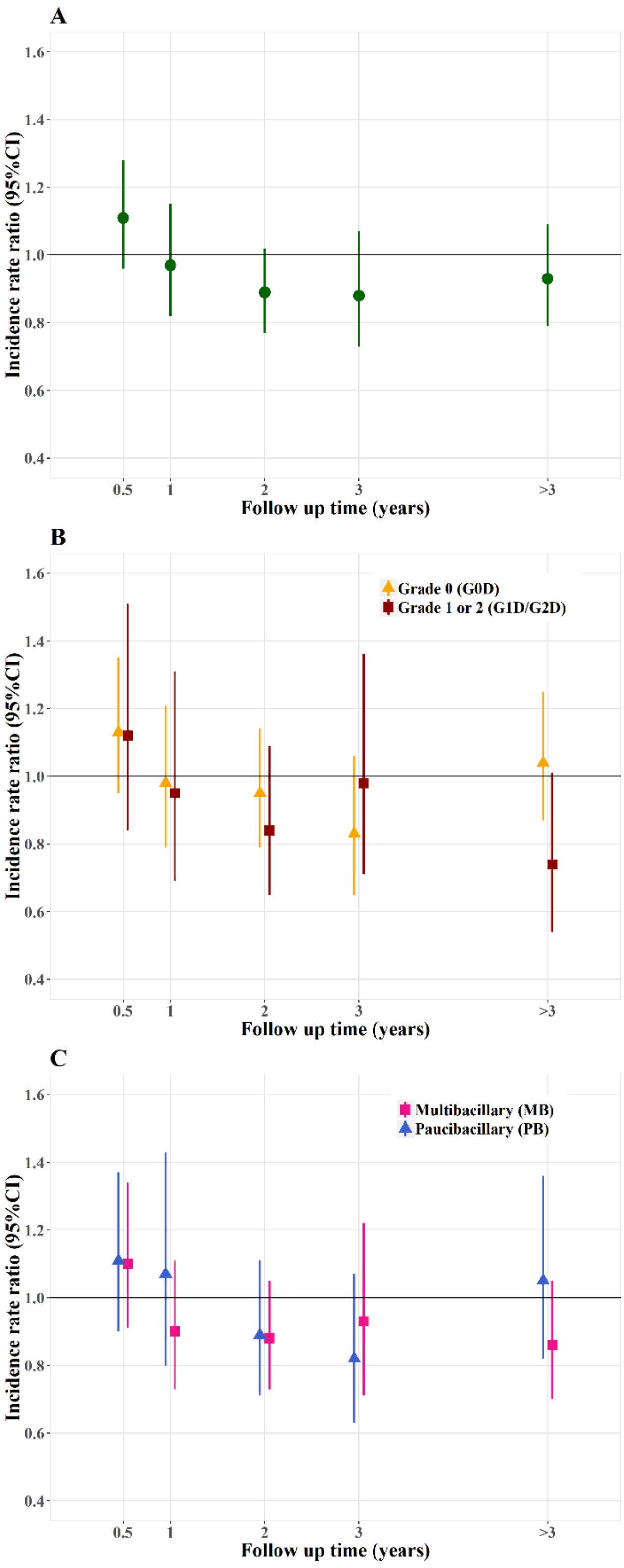

Web Figure 5 IRR of leprosy according to follow up time for the overall matched cohort (A), by disabilities at diagnosis (B) and by leprosy operational classification (C). IRR= incidence rate ratios

(IRR); Follow-up time started after we defined the exposure to BFP benefit. IRR was calculated for each stratified follow-up time (6 months, 1 year, 2 years, 3 years and over 3 years).

Web Table 1 Eligibility criteria and value of benefit received by families included in Bolsa Familia Program (BFP) in Brazil.

	Eligibility for BFP in Brazilian Reais			Benefit in Brazilian Reais			
Year	Extremely Poor	Poor	Date of change	Fixed benefit	Variable A[1]	Variable B[2]	Date of change
2007	R$60	R$120		R$58	R$18	R$30	1st Aug 2007
2008	R$60	R$120		R$62	R$20	R$30	1st Jul 2008
2009	R$70	R$140	1st Sept 2009	R$68	R$22	R$33	1st Sept 2009
2010	R$70	R$140		R$68	R$22	R$33	
2011[3]	R$70	R$140		R$70	R$32	R$38	1st Apr 2011
2012	R$70	R$140		R$70	R$32	R$38	
2013	R$70	R$140		R$70	R$32	R$38	
2014	R$77	R$154	1st Jun 2014	R$77	R$35	R$42	1st Jun 2014

[1]Variable benefit for pregnant woman, breastfeeding, children or adolescents with 15 years old or less.
[2]Variable benefits for grown adolescents at school (16 and 17 years old) - Limit of two per family.
[3]Limit of three variable benefits for pregnant woman, breastfeeding, children or adolescents with 15 years old or less changed to maximum of five. BFP beneficiary families that did not overcome extreme poverty start receiving the necessary amount a month to reach this value.

Web Table 2 New case detection rate (NCDR) of leprosy in Brazil using all cases from the SINAN dataset, estimated and observed number of leprosy cases linked with The 100 Million Brazilian Cohort population.

	Population Brazil 2010*		New leprosy cases (2007-2014)		The 100 Million Brazilian Cohort 2007-2014		Expected number of new leprosy cases linked		Observed number of new leprosy cases linked	
**Variables**	**N**	**%**	**N**	**NCDR/100 000 inhab per year**	**N**	**%**	**N**	**%**	**N**	**%**
**Overall**	190,755,799		282,733	18.5	31,613,355		46,856		44074	
**Sex**										
Female	97,348,809	51.0	126,023	16.2	17,392,677	55.0	22,516	48.05	21,489	48.76
Male	93,406,990	49.0	156,695	21.0	14,220,678	45.0	23,856	50.91	22,581	51.23
Missing		0.0	15	-			-	-	4	0.01
**Age**										
0-9	28,765,533	15	7,434	3.2	10,035,582	31.7	2,594	5.54	1,669	3.79
10-14	17,166,761	9	13,182	9.6	2,345,995	7.4	1,801	3.84	1,946	4.42
15-17	10,357,874	5	8,670	10.5	1,043,584	3.3	874	1.86	1,061	2.41
18-24	23,878,190	13	24,005	12.6	3,261,198	10.3	3,279	7.00	3,618	8.21
25-49	71,580,221	38	124,507	21.7	10,222,256	32.3	17,781	37.95	19,728	44.76
50+	39,007,220	20	104,931	33.6	4,702,939	14.9	12,651	27.00	16,052	36.42
Missing	0	0	4	-	1,801	0.0	-	-	-	-
**Ethnicity**										
White	91,051,646	47.7	81,763	11.2	10,556,910	33.4	9,480	20.23	12,242	27.78
Black	14,517,961	7.6	36,830	31.7	2,003,278	6.3	5,082	10.85	5,756	13.06
Asian	2,084,288	1.1	3,175	19.0	136,788	0.4	208	0.44	498	1.13
Brown/Mixed	82,277,333	43.1	147,101	22.3	17,750,888	56.1	31,736	67.73	23,433	53.17
Indigenous	817,963	0.4	1,170	17.9	232,406	0.7	332	0.71	190	0.43
Missing	6,608	0.0	12,694	-	933,085	3.0	-	-	1,955	4.44
**Area of residence**										
Urban/periurban	160,925,792	84.4	222,360	17.3	25,847,669	81.8	35,715	76.22	34,718	78.77
Rural	29,830,007	15.6	43,459	18.2	5,730,814	18.1	8,349	17.82	6,884	15.62
Missing		0.0	16,914	-	34,872	0.1	-	-	2,472	5.61
**Region**										
North	15,864,454	8.3	56,598	44.6	3,928,431	12.4	14,015	29.91	9,915	22.50
Northeast	53,081,950	27.8	117,612	27.7	9,217,608	29.2	20,423	43.59	16,384	37.17
Southeast	80,364,410	42.1	49,127	7.6	12,184,856	38.5	7,449	15.90	7,354	16.69
South	27,386,891	14.4	11,348	5.2	3,520,309	11.1	1,459	3.11	1,455	3.30
Central-west	14,058,094	7.4	47,956	42.6	2,762,151	8.7	9,422	20.11	8,954	20.32
Missing		0.0	92	-			-	-	12	0.03

Web Table 3 Prediction models using multiple logistic regression for receiving BFP benefit within 6 months after application in The 100 Million Brazilian Cohort for the overall sample of Brazil and in subsamples of high and non-high burden municipalities for leprosy.

	Brazil (overall) adjOR (95%CI)	High-burden municipalities adjOR (95%CI)	Non high-burden municipalities adjOR (95%CI)
**Age (head)**	0.99 (0.99)	0.99 (0.99)	0.99 (0.99)
**Sex (head)**			
Males	1.00	1.00	1.00
Females	0.98 (0.98-0.98)	1.02 (1.02-1.03)	0.95 (0.95-0.96)
**Ethnicity (head)**			
White	1.00	1.00	1.00
Black	1.13 (1.13-1.14)	1.16 (1.15-1.17)	1.10 (1.09-1.11)
Asian	1.00 (0.98-1.02)	0.96 (0.93-0.99)	1.03 (1.00-1.06)
Mixed/brown	1.01 (1.00-1.01)	1.03 (1.02-1.04)	0.99 (0.98-0.99)
Indigenous	5.95 (5.82-6.07)	2.75 (2.62-2.88)	6.39 (6.24-6.55)
Missing	1.02 (1.01-1.03)	1.03 (1.02-1.05)	0.99 (0.98-1.00)
**Literacy (head)**			
Yes	1.00	1.00	1.00
No	1.21 (1.21-1.22)	1.10 (1.09-1.11)	1.29 (1.28-1.3)
Missing	1.21 (1.19-1.23)	1.24 (1.2-1.28)	1.21 (1.19-1.24)
**Education (head)**			
Primary school or less (≤5 years of education)	1.00	1.00	1.00
Junior high school (≤9 years of education)	1.03 (1.03-1.04)	1.07 (1.06-1.08)	1.01 (1.01-1.02)
High school (≥10 years of education)	0.92 (0.91-0.92)	0.98 (0.97-0.98)	0.89 (0.89-0.89)
Missing	0.82 (0.82-0.83)	0.88 (0.87-0.88)	0.79 (0.79-0.8)
**Occupation (head)**			
Unemployed	1.00	1.00	1.00
Employed	0.80 (0.80-0.80)	0.75 (0.74-0.75)	0.83 (0.82-0.83)
Missing	0.99 (0.98-1.00)	0.84 (0.82-0.86)	1.09 (1.07-1.10)
**Individuals per room**	1.37 (1.37-1.37)	1.38 (1.37-1.38)	1.37 (1.36-1.37)
**Indicator of individuals per room missing**			
No	1.00	1.00	1.00
Yes	1.68 (1.6-1.76)	1.80 (1.65-1.97)	1.60 (1.51-1.7)
**Region of residence**			
North	1.00	1.00	1.00
Northeast	0.91 (0.90-0.91)	0.90 (0.89-0.9)	0.87 (0.86-0.88)
Southeast	1.92 (1.91-1.93)	2.19 (2.17-2.21)	1.71 (1.70-1.72)
South	1.44 (1.43-1.45)	1.14 (1.12-1.16)	1.36 (1.35-1.37)
Midwest	1.08 (1.08-1.09)	0.74 (0.73-0.75)	1.39 (1.37-1.40)
**Area of residence**			
Urban	1.00	1.00	1.00
Rural	0.84 (0.84-0.84)	0.63 (0.63-0.64)	0.91 (0.91-0.92)
missing	4.22 (4.05-4.4)	3.89 (3.67-4.13)	4.40 (4.15-4.68)
**Leprosy priority municipality**			
No	1.00	-	-
Yes	1.99 (1.98-1.99)	-	-
**Type of the household**			
Private	1.00	1.00	1.00
Shared and informal housing	1.00 (0.99-1.01)	1.04 (1.02-1.06)	0.94 (0.92-0.95)
missing	0.69 (0.68-0.70)	0.78 (0.76-0.80)	0.62 (0.61-0.63)
**Construction material**			
Bricks/cement	1.00	1.00	1.00
Wood, other vegetal materials and others	0.88 (0.87-0.88)	0.71 (0.70-0.71)	0.95 (0.95-0.96)
missing	0.45 (0.43-0.48)	0.43 (0.39-0.47)	0.50 (0.47-0.53)
**Basic services (water supply, electricicty, sewage and waste)**			
All adequate*	1.00	1.00	1.00
1 inadequate	1.07 (1.07-1.08)	1.06 (1.05-1.06)	1.05 (1.04-1.05)
2 or 3 inadequate	1.15 (1.14-1.15)	1.17 (1.16-1.18)	1.10 (1.09-1.10)
All inadequate	1.08 (1.08-1.09)	1.20 (1.18-1.21)	1.02 (1.01-1.03)
Missing (all)	1.36 (1.35-1.38)	1.33 (1.30-1.36)	1.33 (1.31-1.34)
**Income (percentile)**			
1 (poorest)	1.00	1.00	1.00
2	1.20 (1.19-1.20)	1.39 (1.38-1.40)	1.08 (1.08-1.09)
3	0.83 (0.82-0.83)	0.87 (0.87-0.88)	0.76 (0.76-0.76)
4	0.15 (0.15-0.15)	0.14 (0.14-0.15)	0.15 (0.15-0.16)
5 (richest)	0.05 (0.04-0.05)	0.05 (0.05-0.05)	0.05 (0.05-0.05)
**Year of registry in the cohort**			
2007	1.00	1.00	1.00
2008	0.87 (0.86-0.87)	0.91 (0.90-0.92)	0.82 (0.81-0.82)

2009	4.02 (4.00-4.04)	4.05 (4.01-4.09)	4.06 (4.03-4.09)
2010	2.03 (2.02-2.04)	2.45 (2.42-2.47)	1.79 (1.78-1.80)
2011	3.35 (3.33-3.37)	2.86 (2.83-2.89)	3.59 (3.57-3.62)
2012	1.26 (1.25-1.27)	1.25 (1.23-1.26)	1.26 (1.25-1.27)
2013	1.66 (1.65-1.67)	1.63 (1.61-1.64)	1.60 (1.58-1.61)
2014	2.5 (2.48-2.52)	2.13 (2.1-2.15)	2.69 (2.67-2.72)
**Intercept (beta 0)**	0.61 (0.6-0.62)	1.24 (1.22-1.26)	0.69 (0.68-0.69)

Web Table 4 Standardized mean differences (SMD) between BFP beneficiaries (BFP) and non-beneficiaries (non-BFP) after matching for the overall sample of Brazil and in subsamples of high and non-high-burden municipalities for leprosy.

	Overall			High burden leprosy municipalities			Non-high burden municipalities		
	Non-BFP	BFP		Non-BFP	BFP		Non-BFP	BFP	
	N or mean (% or sd)	N or mean (% or sd)	SMD	N or mean (% or sd)	N or mean (% or sd)	SMD	N or mean (% or sd)	N or mean (% or sd)	SMD
**Age (head)**	35.0 (13.1)	35.3 (12.2)	-0.026	35.4 (13.1)	35.9 (12.0)	-0.042	34.5 (13.1)	34.9 (12.3)	-0.033
**Sex (head)**									
Males	1,799,256 (42.1)	1,677,882 (39.3)	0.058	627,350 (34.1)	576,771 (31.4)	0.059	1,153,784 (47.4)	1,101,065 (45.2)	0.043
Females	2,473,591 (57.9)	2,594,965 (60.7)		1,209,715 (65.9)	1,260,294 (68.6)		1,281,928 (52.6)	1,334,647 (54.8)	
**Ethnicity (head)**									
White	1,171,541 (27.4)	1,199,989 (28.1)	0.019	364,236 (19.8)	384,002 (20.9)	0.034	801,804 (32.9)	815,986 (33.5)	0.018
Black	390,411 (9.1)	384,272 (9.0)		194,267 (10.6)	192,827 (10.5)		193,650 (8.0)	191,443 (7.9)	
Asian	17,172 (0.4)	17,772 (0.4)		8,026 (0.4)	7,991 (0.4)		9,479 (0.4)	9,781 (0.4)	
Mixed/brown	2,469,227 (57.8)	2,438,612 (57.1)		1,177,743 (64.1)	1,153,594 (62.8)		1,302,750 (53.5)	1,285,018 (52.8)	
Indigenous	50,580 (1.2)	50,431 (1.2)		8,673 (0.5)	7,802 (0.4)		39,721 (1.6)	42,562 (1.7)	
Missing	173,916 (4.1)	181,771 (4.3)		84,120 (4.6)	90,849 (4.9)		88,308 (3.6)	90,922 (3.7)	
**Literacy (head)**									
Yes	3,815,014 (89.3)	3,823,908 (89.5)	0.007	1,672,978 (91.1)	1,677,619 (91.3)	0.009	2,143,341 (88.0)	2,146,269 (88.1)	0.004
No	425,748 (10.0)	418,502 (9.8)		150,269 (8.2)	146,201 (8.0)		274,796 (11.3)	272,251 (11.2)	
Missing	32,085 (0.8)	30,437 (0.7)		13,818 (0.8)	13,245 (0.7)		17,575 (0.7)	17,192 (0.7)	
**Education (head)**									
Primary school or less (≤5 years of education)	1,087,268 (25.4)	1,080,076 (25.3)	0.012	395,163 (21.5)	395,993 (21.6)	0.007	687,113 (28.2)	684,061 (28.1)	0.008
Junior high school (≤9 years of education)	1,382,128 (32.3)	1,370,867 (32.1)		605,674 (33.0)	600,537 (32.7)		775,130 (31.8)	770,333 (31.6)	
High school (≥10 years of education)	947,250 (22.2)	967,566 (22.6)		465,092 (25.3)	469,754 (25.6)		490,744 (20.1)	497,808 (20.4)	
Missing	856,201 (20.0)	854,338 (20.0)		371,136 (20.2)	370,781 (20.2)		482,725 (19.8)	483,510 (19.9)	
**Occupation (head)**									

Currently not working	2,013,316 (47.1)	1,967,234 (46.0)	0.023	880,918 (48.0)	859,218 (46.8)	0.025	1,134,301 (46.6)	1,107,989 (45.5)	0.023
Working	1,700,002 (39.8)	1,746,799 (40.9)		688,995 (37.5)	710,079 (38.7)		1,010,293 (41.5)	1,036,677 (42.6)	
Missing	559,529 (13.1)	558,814 (13.1)		267,152 (14.5)	267,768 (14.6)		291,118 (12.0)	291,046 (11.9)	
**Rooms per capita**	0.96 (0.96)	0.93 (0.75)	0.036	1.03 (1.00)	1.00 (0.77)	0.038	0.91 (0.93)	0.89 (0.73)	0.029
**Indicator of rooms per capita missing**									
No	4,151,318 (97.2)	4,144,095 (97.0)	0.010	1,784,340 (97.1)	1,780,583 (96.9)	0.012	2,366,619 (97.2)	2,363,441 (97.0)	0.008
Yes	121,529 (2.8)	128,752 (3.0)		52,725 (2.9)	56,482 (3.1)		69,093(2.8)	72,271 (3.0)	
**Region of residence**									
North	596,121 (14.0)	555,511 (13.0)	0.035	363,755 (19.8)	327,342 (17.8)	0.059	238,648 (9.8)	228,156 (9.4)	0.022
Northeast	1,316,574 (30.8)	1,330,342 (31.1)		594,600 (32.4)	603,329 (32.8)		733,880 (30.1)	727,013 (29.8)	
Southeast	1,703,535 (39.9)	1,735,547 (40.6)		713,181 (38.8)	744,363 (40.5)		991,568 (40.7)	99,1180 (40.7)	
South	339,164 (7.9)	351,872 (8.2)		42,055 (2.3)	46,996 (2.6)		292,239 (12.0)	304,874 (12.5)	
Midwest	317,453 (7.4)	299,575 (7.0)		123,474 (6.7)	115,035 (6.3)		179,377 (7.4)	184,489 (7.6)	
**Area of residence**									
Urban	3,494,458 (81.8)	3,499,106 (81.9)	0.008	1,708,296 (93.0)	1,708,899 (93.0)	0.014	1,787,244 (73.4)	1,790,200 (73.5)	0.006
Rural	764,170 (17.9)	757,694 (17.7)		119,723 (6.5)	117,377 (6.4)		643,767 (26.4)	640,253 (26.3)	
missing	14,219 (0.3)	16,047 (0.4)		9,046 (0.5)	10,789 (0.6)		4,701 (0.2)	5,259 (0.2)	
**Leprosy priority municipality**									
No	2,437,360 (57.0)	2,435,829 (57.0)	0.001						
Yes	1,835,487 (43.0)	1,837,018 (43.0)							
**Type of the household**									
Private	3,598,216 (84.2)	3,579,482 (83.8)	0.012	1,507,327 (82.1)	1497458 (81.5)	0.014	2091544 (85.9)	2,081,945 (85.5)	0.011
Shared and informal housing	166,511 (3.9)	172,573 (4.0)		77,561 (4.2)	79089 (4.3)		90310 (3.7)	93,490 (3.8)	
missing	508,120 (11.9)	520,792 (12.2)		252,177 (13.7)	260518 (14.2)		253858 (10.4)	260,277 (10.7)	
**Construction material**									
Bricks/cement	3,332,186 (78.0)	3,355,920 (78.5)	0.021	1,505,842 (82.0)	1,520,516 (82.8)	0.030	1,831,646 (75.2)	1,835,388 (75.4)	0.010
Wood, other vegetal materials and others	820,152 (19.2)	788,831 (18.5)		279,158 (15.2)	260,654 (14.2)		535,080 (22.0)	528,122 (21.7)	
missing	120,509 (2.8)	128,096 (3.0)		52,065 (2.8)	55,895 (3.0)		68,986 (2.8)	72,202 (3.0)	

**Basic services (water supply, electricicty, sewage and waste)**									
All adequate*	2,089,827 (48.9)	2,108,816 (49.4)	0.017	945,043 (51.4)	971,307 (52.9)	0.035	1,136,661 (46.7)	1,137,493 (46.7)	0.014
1 inadequate	930,043 (21.8)	928,179 (21.7)		447,321 (24.3)	442,894 (24.1)		481,874 (19.8)	485,287 (19.9)	
2 or 3 inadequate	496,480 (11.6)	483,586 (11.3)		218,414 (11.9)	204,390 (11.1)		280,413 (11.5)	279,189 (11.5)	
All inadequate	517,966 (12.1)	504,301 (11.8)		142,204 (7.7)	133,225 (7.3)		379,846 (15.6)	371,054 (15.2)	
Missing (all)	238,531 (5.6)	247,965 (5.8)		84,083 (4.6)	85,249 (4.6)		156,918 (6.4)	162,689 (6.7)	
**Income (percentile)**									
1 (poorest)	1,252,472 (29.3)	1,221,092 (28.6)	0.034	522,541 (28.4)	505,188 (27.5)	0.029	743,584 (30.5)	734,417 (30.2)	0.025
2	1,317,394 (30.8)	1,368,642 (32.0)		561,256 (30.6)	573,752 (31.2)		763,163 (31.3)	776,304 (31.9)	
3	1,154,675 (27.0)	1,168,007 (27.3)		517,033 (28.1)	530,415 (28.9)		644,362 (26.5)	652,347 (26.8)	
4	440,364 (10.3)	411,903 (9.6)		175,493 (9.6)	170,230 (9.3)		228,345 (9.4)	213,545 (8.8)	
5 (richest)	107,942 (2.5)	103,203 (2.4)		60,742 (3.3)	57,480 (3.1)		56,258 (2.3)	59,099 (2.4)	
**Year of application**									
2007	639,034 (15.0)	606,955 (14.2)	0.065	239,187 (13.0)	227,075 (12.4)	0.073	400,554 (16.4)	379,883 (15.6)	0.060
2008	399,496 (9.3)	395,917 (9.3)		186,468 (10.2)	186,841 (10.2)		217,732 (8.9)	209,074 (8.6)	
2009	834,707 (19.5)	768,792 (18.0)		295,545 (16.1)	281,252 (15.3)		515,200 (21.2)	487,521 (20.0)	
2010	688,217 (16.1)	662,685 (15.5)		369,885 (20.1)	336,198 (18.3)		337,749 (13.9)	326,485 (13.4)	
2011	498,123 (11.7)	528,465 (12.4)		184,739 (10.1)	201,479 (11.0)		302,745 (12.4)	326,980 (13.4)	
2012	526,404 (12.3)	551,427 (12.9)		248,618 (13.5)	257,772 (14.0)		285,519 (11.7)	293,642 (12.1)	
2013	437,969 (10.3)	483,970 (11.3)		205,029 (11.2)	226,670 (12.3)		237,917 (9.8)	257,288 (10.6)	
2014	248,897 (5.8)	274,636 (6.4)		107,594 (5.9)	119,778 (6.5)		138,296 (5.7)	154,839 (6.4)	

Web Table 5 Incidence rate ratio (IRR) of leprosy for exposure to BFP according to duration of BFP benefits for families residing in municipalities of high or non-high leprosy burden. IRR estimated using Poisson regression further adjusting for income. IRR=Incidence rate ratio; BFP=Bolsa Familia Program.

	**New leprosy cases**	**IRR[2] (95%CI) by time after BFP-receipt**				
		**0.5 years**	**1 year**	**2 years**	**3 years**	**>3 years**
**Overall matched cohort (nº families= 8,545,694) [3]**						
**All new cases**	9,886	1.11 (0.96-1.28)	0.97 (0.82-1.15)	0.89 (0.77-1.02)	0.88 (0.73-1.07)	0.93 (0.79-1.09)
**Grade 0**	6,371	1.13 (0.95-1.35)	0.98 (0.79-1.21)	0.95 (0.79-1.14)	0.83 (0.65-1.06)	1.04 (0.87-1.25)
**Grade 1 or 2**	2,534	1.12 (0.84-1.51)	0.95 (0.69-1.31)	0.84 (0.65-1.09)	0.98 (0.71-1.36)	0.74 (0.54-1.01)
**Paucibacillary cases (PB)**	4,022	1.11 (0.90-1.37)	1.07 (0.80-1.43)	0.89 (0.71-1.11)	0.82 (0.63-1.07)	1.05 (0.82-1.36)
**Multibacillary cases (MB)**	5,860	1.10 (0.91-1.34)	0.90 (0.73-1.11)	0.88 (0.73-1.05)	0.93 (0.71-1.22)	0.86 (0.70-1.05)
**High-burden municipalities (nº families= 3,674,130) [4]**						
**All new cases**	5,394	0.75 (0.59-0.95)	0.87 (0.69-1.10)	0.89 (0.72-1.10)	0.87 (0.65-1.16)	0.93 (0.73-1.17)
**Grade 0**	3,620	0.92 (0.71-1.21)	0.84 (0.63-1.12)	0.92 (0.71-1.19)	0.84 (0.60-1.17)	1.02 (0.73-1.42)
**Grade 1 or 2**	1,251	0.70 (0.46-1.07)	1.00 (0.65-1.55)	0.70 (0.47-1.05)	0.92 (0.47-1.78)	0.76 (0.55-1.05)
**Paucibacillary cases (PB)**	2,415	0.64 (0.44-0.93)	0.85 (0.60-1.22)	0.86 (0.62-1.20)	0.72 (0.47-1.12)	1.27 (0.88-1.83)
**Multibacillary cases (MB)**	2,978	0.86 (0.65-1.14)	0.88 (0.66-1.19)	0.91 (0.70-1.19)	1.01 (0.69-1.48)	0.76 (0.57-1.02)
**Non high burden municipalities (nº families= 4,871,424) [5]**						
**All new cases**	4,578	1.09 (0.88-1.36)	0.89 (0.72 (1.10)	0.95 (0.79-1.15)	1.10 (0.84-1.43)	0.88 (0.73-1.06)
**Grade 0**	2,746	1.04 (0.78-1.38)	0.96 (0.73-1.27)	1.06 (0.82-1.36)	1.41 (0.96-2.07)	0.86 (0.67-1.11)
**Grade 1 or 2**	1,319	1.23 (0.83-1.83)	0.71 (0.48-1.05)	0.95 (0.68-1.33)	0.82 (0.52-1.32)	1.00 (0.72-1.40)
**Paucibacillary cases (PB)**	1,672	0.98 (0.71-1.35)	1.10 (0.74-1.65)	1.09 (0.79-1.49)	1.53 (1.03-2.29)	0.83 (0.62-1.12)
**Multibacillary cases (MB)**	2,903	1.17 (0.87-1.58)	0.79 (0.61-1.02)	0.89 (0.70-1.12)	0.93 (0.66-1.30)	0.91 (0.71-1.16)

[1]Family-level person years at risk
[2]Incidence rate ratio estimated using Poisson regression adjusting for income (continuous) and including robust standard errors clustered by family.
[3]In the stratified analysis, missing in grade of disabilities at diagnosis (N=981) or operational classification (N=4) were censored at the time the leprosy case occurred.
[4] In the stratified analysis, missing in grade of disabilities at diagnosis (N=523) or operational classification (N=1) were censored at the time the leprosy case occurred.
[5] In the stratified analysis, missing in grade of disabilities at diagnosis (N=513) or operational classification (N=3) were censored at the time the leprosy case occurred.

Web Table 6 Incidence rate ratio (IRR) of leprosy (overall and according to grade of disabilities and operational classification) for BFP participation, in the matched cohorts considering the BFP treatment to vary over time: Overall sample, high and lower leprosy burden municipalities.

**Brazil**<br>families= 13,506,522;<br>fyr[1]=37,243,946.5; pyr=102,760,538	**Leprosy cases**	**IR in BFP**	**IR in non-BFP**	**IRR[2] (95%CI)**	**IRR[3] (95%CI)**
**All new cases[4]**	16,416	15.77 (15.49-16.06)	16.48 (16.03-16.96)	0.96 (0.93-0.99)	0.97 (0.91-1.03)
**Grade 0**	10,484	10.19 (9.96-10.42)	10.23 (9.87-10.61)	0.99 (0.95-1.04)	1.00 (0.93-1.08)
**Grade 1 or 2 disabilities**	4,244	3.98 (3.84-4.13)	4.50 (4.27-4.75)	0.88 (0.83-0.94)	0.90 (0.81-1.00)
**Paucibacillary cases (PB)**	6,662	6.50 (6.31-6.68)	6.45 (6.16-6.75)	1.01 (0.96-1.06)	1.00 (0.92-1.10)
**Multibacillary cases (MB)**	9,747	9.27 (9.05-9.49)	10.03 (9.67-10.40)	0.92 (0.88-0.97)	0.94 (0.87-1.02)
**Leprosy high-burden municipalities**<br>families= 5,073,770;<br>fyr[1]= 13,460,216; pyr=37,262,080.2	**Leprosy cases**	**IR in BFP**	**IR in non-BFP**	**IRR[2] (95%CI)**	**IRR[3] (95%CI)**
**All new cases[5]**	8,305	21.60 (21.06-22.16)	24.15 (23.20-25.14)	0.89 (0.85-0.94)	0.90 (0.82-0.99)
**Grade 0**	5,680	14.96 (14.51-15.43)	16.01 (15.25-16.82)	0.93 (0.88-0.99)	0.93 (0.84-1.04)
**Grade 1 or 2 disabilities**	1,854	4.72 (4.46-4.98)	5.68 (5.24-6.17)	0.83 (0.75-0.92)	0.85 (0.72-1.01)
**Paucibacillary cases (PB)**	3,761	9.81 (9.44-10.18)	10.88 (10.25-11.54)	0.90 (0.84-0.97)	0.89 (0.77-1.03)
**Multibacillary cases (MB)**	4,542	11.79 (11.39-12.21)	13.28 (12.58-14.01)	0.89 (0.83-0.95)	0.90 (0.80-1.01)
**Leprosy low-burden municipalities**<br>families= 8,432,536;<br>fyr[1]=23,734,819.9; pyr=65,309,408.4	**Leprosy cases**	**IR in BFP**	**IR in non-BFP**	**IRR[2] (95%CI)**	**IRR[3] (95%CI)**
**All new cases[6]**	8,140	12.34 (12.02-12.66)	12.77 (12.27-13.29)	0.97 (0.92-1.01)	0.98 (0.90-1.05)
**Grade 0**	4,796	7.38 (7.14-7.63)	7.25 (6.88-7.64)	1.02 (0.96-1.08)	1.02 (0.93-1.14)
**Grade 1 or 2 disabilities**	2,409	3.55 (3.38-3.72)	4.03 (3.75-4.33)	0.88 (0.81-0.96)	0.89 (0.78-1.03)
**Paucibacillary cases (PB)**	2,968	4.55 (4.36-4.75)	4.53 (4.24-4.85)	1.00 (0.93-1.09)	1.00 (0.88-1.13)
**Multibacillary cases (MB)**	5,167	7.78 (7.53-8.04)	8.23 (7.83-8.65)	0.95 (0.89-1.00)	0.96 (0.87-1.06)

[1] Family-level person years at risk

[2] Incidence rate ratio estimated estimated using Mantel-Haenszel method.

[3] Incidence rate ratio estimated using Poisson regression adjusting for income (continuous) and including robust standard errors clustered by family.

4 In the stratified analysis, missing in grade of disabilities at diagnosis (N=1688) or operational classification (N=7) were censored at the time the leprosy case occurred.

5 In the stratified analysis, missing in grade of disabilities at diagnosis (N=771) or operational classification (N=2) were censored at the time the leprosy case occurred.

6 In the stratified analysis, missing in grade of disabilities at diagnosis (N=925) or operational classification (N=5) were censored at the time the leprosy case occurred.

Web Table 7 Incidence rate ratio (IRR) of leprosy for exposure to BFP overall and according to duration of BFP benefits in the overall matched cohort (N= 8,545,694 families). IRR estimated using Poisson regression without adjusting for income and adjusted for income as cubic splines. IRR=Incidence rate ratio; BFP=Bolsa Familia Program.

fyr[1]= 23,467,162.1; pyr=65,878,418.7	**Overall IRR (95%CI)[3]**	**IRR[2] (95%CI) by time after BFP-receipt**				
		**0.5 years**	**1 year**	**2 years**	**3 years**	**>3 years**
**Without additional controlling for income**						
**All new cases**	0.96 (0.89-1.03)	1.10 (0.95-1.27)	0.96 (0.81-1.14)	0.87 (0.76-1.00)	0.87 (0.72-1.05)	0.92 (0.78-1.07)
**Grade 0**	0.99 (0.91-1.08)	1.12 (0.94-1.34)	0.97 (0.78-1.19)	0.94 (0.78-1.12)	0.81 (0.64-1.04)	1.03 (0.85-1.23)
**Grade 1 or 2**	0.91 (0.79-1.03)	1.11 (0.83-1.50)	0.94 (0.68-1.30)	0.83 (0.64-1.07)	0.96 (0.69-1.34)	0.73 (0.54-1.00)
**Paucibacillary cases (PB)**	0.99 (0.89-1.10)	1.11 (0.90-1.38)	1.08 (0.81-1.44)	0.89 (0.72-1.11)	0.82 (0.63-1.07)	1.06 (0.82-1.36)
**Multibacillary cases (MB)**	0.94 (0.86-1.03)	1.09 (0.90-1.32)	0.88 (0.72-1.09)	0.86 (0.72-1.03)	0.91 (0.69-1.19)	0.84 (0.69-1.03)
**Additionally controlling for income as spline**						
**All new cases**	0.93 (0.87-1.00)	1.10 (0.95-1.27)	0.95 (0.80-1.13)	0.85 (0.74-0.98)	0.83 (0.68-1.01)	0.86 (0.74-1.01)
**Grade 0**	0.96 (0.88-1.05)	1.12 (0.94-1.33)	0.95 (0.77-1.18)	0.91 (0.76-1.09)	0.77 (0.60-0.99)	0.96 (0.79-1.15)
**Grade 1 or 2**	0.91 (0.79-1.05)	1.12 (0.84-1.51)	0.95 (0.69-1.31)	0.83 (0.64-1.08)	0.97 (0.70-1.34)	0.73 (0.53-0.99)
**Paucibacillary cases (PB)**	0.93 (0.83-1.04)	1.09 (0.89-1.35)	1.04 (0.78-1.40)	0.84 (0.68-1.05)	0.75 (0.57-0.98)	0.95 (0.73-1.23)
**Multibacillary cases (MB)**	0.93 (0.85-1.02)	1.09 (0.90-1.33)	0.89 (0.72-1.09)	0.86 (0.72-1.03)	0.90 (0.68-1.18)	0.82 (0.67-1.00)

1Family-level person years at risk
2Incidence rate ratio estimated using Poisson regression including robust standard errors clustered by family.
3In the stratified analysis, missing in grade of disabilities at diagnosis or operational classification were censored at the time the leprosy case occurred.

Web Table 8 IRR of having leprosy according to exposure to BFP overall and according to duration of BFP benefits among families using Inverse Probability of the Treatment Weighting (IPTW) (N=12,037,799 families). IRR estimated using Poisson regression further adjusting for income. IRR=Incidence rate ratio; BFP=Bolsa Familia Program.

fyr[1]= 32.590.798.4; pyr=82,108,674.4	**New leprosy cases[3]**	**Overall IRR[2] (95%CI)**	**IRR[2] (95%CI) by time after BFP-receipt**				
			**0.5 years**	**1 year**	**2 years**	**3 years**	**>3 years**
**All new cases**	14,309	0.95 (0.90-1.00)	0.93 (0.83-1.06)	0.93 (0.82-1.06)	0.91 (0.82-1.01)	0.96 (0.84-1.10)	0.97 (0.88-1.07)
**Grade 0**	8,777	1.00 (0.94-1.06)	1.13 (0.92-1.37)	0.81 (0.65-1.02)	0.96 (0.82-1.14)	0.97 (0.81-1.17)	1.03 (0.94-1.14)
**Grade 1 or 2**	4,098	0.87 (0.79-0.96)	0.94 (0.69-1.29)	1.01 (0.74-1.39)	0.95 (0.74-1.21)	0.78 (0.59-1.04)	0.89 (0.76-1.05)
**Paucibacillary cases (PB)**	5,375	0.94 (0.86-1.02)	0.88 (0.62-1.24)	0.78 (0.57-1.08)	0.98 (0.79-1.20)	0.88 (0.69-1.13)	1.00 (0.88-1.13)
**Multibacillary cases (MB)**	8,924	0.96 (0.90-1.02)	1.02 (0.83-1.25)	0.95 (0.77-1.19)	0.94 (0.80-1.12)	0.88 (0.73-1.06)	1.00 (0.90-1.12)

[1]Family-level person years at risk.
[2]Incidence rate ratio estimated using Poisson regression adjusting for income (continuous) and weighting for the inverse probability of receiving the treatment. We included cluster-robust standard errors by family only for the full cohort analysis.
[3] In the stratified analysis, missing in grade of disabilities at diagnosis (N=1,434) or operational classification (N=10) were censored at the time the leprosy case occurred.

Web Table 9 Incidence rate ratio (IRR) of leprosy for exposure to BFP overall and according to duration of BFP benefits in the overall matched cohort only for families without missing data in matching variables (i.e., complete cases analysis) (N=5,391,086 families).

fyr[1]=14677939.1; pyr=42,602,148.7	**Leprosy cases[3]**	**Overall IRR[2] (95%CI)**	**IRR[2] (95%CI) by time after BFP-receipt**				
			**0.5 years**	**1 year**	**2 years**	**3 years**	**>3 years**
**All new cases**	6,255	0.99 (0.90-1.10)	0.93 (0.73-1.19)	1.02 (0.83-1.26)	0.89 (0.75-1.05)	0.89 (0.67-1.19)	1.22 (1.01-1.48)
**Grade 0**	4,037	1.06 (0.94-1.18)	1.15 (0.92-1.44)	1.07 (0.83-1.37)	0.94 (0.76-1.17)	0.85 (0.59-1.22)	1.21 (0.96-1.54)
**Grade 1 or 2**	1,570	0.94 (0.80-1.12)	1.01 (0.70-1.45)	0.92 (0.60-1.43)	0.77 (0.56-1.06)	0.92 (0.55-1.59)	1.09 (0.75-1.59)
**Paucibacillary cases (PB)**	2,686	0.89 (0.74-1.06)	0.87 (0.55-1.39)	1.10 (0.78-1.55)	0.87 (0.66-1.13)	0.68 (0.44-1.08)	0.97 (0.73-1.30)
**Multibacillary cases (MB)**	3,566	1.08 (0.96-1.20)	0.98 (0.78-1.24)	0.97 (0.75-1.25)	0.90 (0.72-1.12)	1.14 (0.81-1.59)	1.45 (1.13-1.87)

[1]Family-level person years at risk
[2]Incidence rate ratio estimated using Poisson regression adjusting for income (continuous) and including robust standard errors clustered by family.
[3]In the stratified analysis, missing in grade of disabilities at diagnosis (N=648) or operational classification (N=3) were censored at the time the leprosy case occurred.

Web Table 10 Incidence rate ratio (IRR) of leprosy for exposure to BFP overall and according to grade of disabilities and operational classification in the matched cohorts after restricting fyr to the smallest between each matched pair: Overall sample (N=8,545,694 families), high (i.e., N=3,674,130 families) and non-high leprosy burden municipalities (N=4,871,424 families).

	**Brazil** fyr[1]=11341667; pyr=30,357,816.3		**Leprosy priority municipalities** fyr[1]= 4,544,053; pyr=12,071,588		**Non-priority municipalities** fyr[1]=6,801,939; pyr=18,237,139.7	
	**Leprosy cases**	**IRR[2] (95%CI)**	**Leprosy cases**	**IRR[2] (95%CI)**	**Leprosy cases**	**IRR[2] (95%CI)**
**All new cases**	4,650	0.99 (0.91-1.07)	2,485	0.85 (0.75-0.97)	2,180	1.02 (0.91-1.14)
**Grade 0[3]**	3,009	1.02 (0.93-1.13)	1,657	0.91 (0.78-1.05)	1,284	1.08 (0.93-1.25)
**Grade 1 or 2[3]**	1,098	0.96 (0.82-1.12)	579	0.81 (0.64-1.03)	649	0.96 (0.79-1.17)
**Paucibacillary cases (PB)[3]**	1,865	1.00 (0.89-1.14)	1,122	0.76 (0.62-0.94)	812	1.09 (0.91-1.30)
**Multibacillary cases (MB)[3]**	2,783	0.98 (0.88-1.09)	1,363	0.94 (0.80-1.10)	1,367	0.98 (0.85-1.12)

[1]Family-level person years at risk
[2]Incidence rate ratio estimated using Poisson regression adjusting for income (continuous) and including cluster-robust standard errors by family.
[3]In the stratified analysis, missing in grade of disabilities at diagnosis or operational classification were censored at the time the leprosy case occurred.

Web Table 11 Incidence rate ratio (IRR) of leprosy for exposure to BFP overall and according to grade of disabilities and operational classification in the matched cohorts after restricting fyr to 2 years: Overall sample (N=8,545,694 families), high (i.e., N=3,674,130 families) and non-high leprosy burden municipalities (N=4,871,424families).

	**Brazil** fyr[1]=12,230,323.9; pyr=33,972,802.3		**Leprosy priority municipalities** fyr[1]= 5,133,691; pyr=14,265,231.1		**Non-priority municipalities** fyr[1]=7,079,572; pyr=19,618,975.8	
	**Leprosy cases**	**IRR[2] (95%CI)**	**Leprosy cases**	**IRR[2] (95%CI)**	**Leprosy cases**	**IRR[2] (95%CI)**
**All new cases**	4,927	0.98 (0.90-1.07)	2,808	0.81 (0.71-0.93)	2,255	0.98 (0.87-1.10)
**Grade 0[3]**	3,157	1.02 (0.91-1.13)	1,886	0.89 (0.76-1.04)	1,314	1.02 (0.88-1.20)
**Grade 1 or 2[3]**	1,283	0.95 (0.80-1.13)	642	0.75 (0.58-0.97)	696	0.95 (0.76-1.18)
**Paucibacillary cases (PB)[3]**	2,047	1.01 (0.88-1.16)	1,301	0.75 (0.60-0.94)	848	1.03 (0.84-1.26)
**Multibacillary cases (MB)[3]**	2,878	0.95 (0.85-1.07)	1,507	0.87 (0.74-1.03)	1,405	0.94 (0.81-1.10)

[1]Family-level person years at risk
[2]Incidence rate ratio estimated using Poisson regression adjusting for income (continuous) and including cluster-robust standard errors by family.
[3]In the stratified analysis, missing in grade of disabilities at diagnosis or operational classification were censored at the time the leprosy case occurred.

References

1. Barreto ML, Ichihara MYT, Almeida BdA, Barreto ME, Cabral L, Fiaccone RL, et al. The Centre for Data and Knowledge Integration for Health (CIDACS): Linking Health and Social Data in Brazil. International Journal of Population Data Science. 2019;4(2):04.
2. Ali MS, Ichihara MY, Lopes LC, Barbosa GC, Pita R, Carreiro RP, et al. Administrative data linkage in Brazil: potentials for health technology assessment. Front Pharmacol. 2019;10.
3. Pescarini JM, Alves A, Strina A, Cidacs. Dataset - Leprosy incidence and treatment outcomes in the 100 Million Brazilian Cohort. 2019. https://hdl.handle.net/20.500.12196/FK2/FNMRCA.
4. Direito DC, Koga NM, Licio EC, Chaves JCPN. O Cadastro Único como instrumento de articulação de políticas sociais. Brasilia: United Nations Development Programme (UNDP); 2016.
5. Campello T, Neri MC. Programa Bolsa Família: uma década de inclusão e cidadania. *Brasília: Ipea* 2013.
6. Galvao PR, Ferreira AT, Maciel MD, et al. An evaluation of the Sinan health information system as used by the Hansen's disease control programme, Pernambuco State, Brazil. *Leprosy review* 2008; **79**(2): 171-82.
7. Pita R, Pinto C, Sena S, et al. On the Accuracy and Scalability of Probabilistic Data Linkage Over the Brazilian 114 Million Cohort. *IEEE J Biomed Health Inform* 2018; **22**(2): 346-53.
8. IBGE. Censo Demografico 2010. Características da população e dos domicílios: resultados do universo. Rio de Janeiro: IBGE; 2011.
